# The Interactomes of Influenza Virus NS1 and NS2 Proteins Identify New Host Factors and Provide Insights for ADAR1 Playing a Supportive Role in Virus Replication

**DOI:** 10.1371/journal.ppat.1003440

**Published:** 2013-07-04

**Authors:** Benoît de Chassey, Anne Aublin-Gex, Alessia Ruggieri, Laurène Meyniel-Schicklin, Fabrine Pradezynski, Nathalie Davoust, Thibault Chantier, Lionel Tafforeau, Philippe-Emmanuel Mangeot, Claire Ciancia, Laure Perrin-Cocon, Ralf Bartenschlager, Patrice André, Vincent Lotteau

**Affiliations:** 1 Hospices Civils de Lyon, Hôpital de la Croix Rousse, Laboratory of Virology, Lyon, France; 2 CIRI, International Center for Infectiology Research, EVIR Team, Université de Lyon, Lyon, France; 3 Inserm, U1111, Lyon, France; 4 Ecole Normale Supérieure de Lyon, Lyon, France; 5 Université Lyon 1, Centre International de Recherche en Infectiologie, Lyon, France; 6 CNRS, UMR5308, Lyon, France; 7 Department for Infectious Diseases, Molecular Virology, University of Heidelberg, Heidelberg, Germany; Burnham Institute for Medical Research, United States of America

## Abstract

Influenza A NS1 and NS2 proteins are encoded by the RNA segment 8 of the viral genome. NS1 is a multifunctional protein and a virulence factor while NS2 is involved in nuclear export of viral ribonucleoprotein complexes. A yeast two-hybrid screening strategy was used to identify host factors supporting NS1 and NS2 functions. More than 560 interactions between 79 cellular proteins and NS1 and NS2 proteins from 9 different influenza virus strains have been identified. These interacting proteins are potentially involved in each step of the infectious process and their contribution to viral replication was tested by RNA interference. Validation of the relevance of these host cell proteins for the viral replication cycle revealed that 7 of the 79 NS1 and/or NS2-interacting proteins positively or negatively controlled virus replication. One of the main factors targeted by NS1 of all virus strains was double-stranded RNA binding domain protein family. In particular, adenosine deaminase acting on RNA 1 (ADAR1) appeared as a pro-viral host factor whose expression is necessary for optimal viral protein synthesis and replication. Surprisingly, ADAR1 also appeared as a pro-viral host factor for dengue virus replication and directly interacted with the viral NS3 protein. ADAR1 editing activity was enhanced by both viruses through dengue virus NS3 and influenza virus NS1 proteins, suggesting a similar virus-host co-evolution.

## Author Summary

Viruses are obligate intracellular parasites that rely on cellular functions for efficient replication. As most biological processes are sustained by protein-protein interactions, the identification of interactions between viral and host proteins can provide a global overview about the cellular functions engaged during viral replication. Influenza viruses express 13 viral proteins, including NS1 and NS2, which are translated from an alternatively spliced RNA derived from the same genome segment. We present here a comprehensive overview of possible interactions of cellular proteins with NS1 and NS2 from 9 viral strains. Seventy nine cellular proteins were identified to interact with NS1, NS2 or both NS1 and NS2. These interacting host cell proteins are potentially involved in many steps of the virus life cycle and 7 can directly control the viral replication. Most of the cellular targets are shared by the majority of the virus strains, especially the double-stranded RNA binding domain protein family that is strikingly targeted by NS1. One of its members, ADAR1, is essential for influenza virus replication. ADAR1 colocalizes with NS1 in nuclear structures and its editing activity is enhanced by NS1 expressed on its own and during virus infection. A similar phenomenon is observed for dengue virus whose NS3 protein also interacts with ADAR1, suggesting a parallel virus-host co-evolution.

## Introduction

Influenza A viruses are the causative agents of seasonal and pandemic infections and are responsible for the death of at least half a million people worldwide each year. The genome of influenza A viruses is composed of eight negative-sense single-stranded RNAs encoding 13 proteins. NS1 and NS2 are derived from alternatively spliced RNAs that are transcribed from the eighth RNA segment. The segments are encapsidated by binding to nucleoproteins (NP) and the polymerase complex (PA, PB1 and PB2) forming the viral ribonucleoproteins (vRNPs). The viral particle contains eight vRNPs, the surface glycoproteins haemagglutinin (HA) and neuraminidase (NA), the matrix proteins (M1 and M2) and the NS2 protein. Some strains express the pro-apoptotic PB1-F2 protein and two additional virulence factors, PB1-N40 and PA-X, have been recently identified [1]–[3].

The NS1 protein is not incorporated in the virus. It exerts a large spectrum of functions through interactions with a variety of cellular components residing either in the cytoplasm or in the nucleus. NS1 is a pleiotropic virulence factor repressing innate antiviral mechanisms *e.g.* by interfering with the type I interferon system through direct interaction with PKR and TRIM25, or through the sequestration of double-stranded RNA [4]–[8]. NS1 is also known to perturb the mRNA processing by interacting with CPSF4 and PABPN1 to inhibit nuclear export of cellular mRNA [9] and is suspected to hijack the RNA translation machinery in favor of translation of viral protein *e.g.* by interacting with STAU1 [10]–[11].

In contrast to NS1, NS2 protein is a structural component of the viral particle and it associates with the viral matrix M1 protein [12]. NS2 mediates the export of vRNPs from the nucleus to the cytoplasm through export signal [13] via its interaction with XPO1 [14]. In addition, NS2 interacts with nucleoporins and was suggested to serve as an adaptor between vRNPs and the nuclear pore complex [13]. A role of NS2 in the regulation of influenza virus transcription and replication has also been proposed [15]. However, many functions of NS2, in particular its transit through the cytoplasm and its incorporation into the viral particle, are not understood.

Several screens have been performed to identify host factors involved in the influenza virus replication cycle, mainly focusing on interactors of vRNPs or of the polymerase by using affinity purification or yeast two-hybrid techniques [16]–[18]. A proteome-wide screen of virus-host protein-protein interactions has provided an important resource of 135 interactions [19]. However, the weak overlap of the public datasets suggests that they are far from being complete.

The impact of cellular proteins on the influenza virus replication has been extensively studied using RNAi screens [19]–[24]. Although poorly overlapping at the gene level, these screens better converge at the level of biological processes [25]–[27]. Hence, more than the identification of host factors, these studies highlighted major cellular functions that are essential for the virus replication. However, for the majority of identified host factors, the mode of action remains to be determined. Furthermore, comparisons of strain-specific virus-host interactomes are clearly missing, which is required to reveal general principles governing infection mechanisms and to identify common therapeutic targets as well as broad-spectrum antivirals.

In the present study we conducted stringent yeast two-hybrid screens to identify human proteins interacting with NS1 and NS2 from 9 influenza A virus strains representative of the variability in nature. The functional impact of all NS1 and NS2 interactors on viral replication was systematically addressed by RNA interference. In combination with published datasets, our new results offer a comprehensive view of NS1 and NS2 interactomes and corresponding targeted cellular functions. The global analysis of the NS1 and NS2 host cell targets reveals an enrichment of double-stranded RNA binding domain (DRBD) containing proteins for the 9 tested influenza virus strains. A focus was put on ADAR1 since this protein is critical for the replication of other viruses [28], is highly expressed in human lung cells [29], is induced by type I interferon [30], is interfering with interferon signalling production [31] and is interacting with all tested NS1 proteins. In addition, we also observed in another screen that ADAR1 interacts with the dengue virus NS3 protein which is a bifunctional enzyme containing protease and helicase activity [32]. We show that ADAR1 is a pro-viral host factor favoring replication of influenza virus and dengue virus and that these viral proteins can control ADAR1 editing activity.

## Results

### Cellular interactors of influenza virus NS1 and NS2

**Figure 1:**
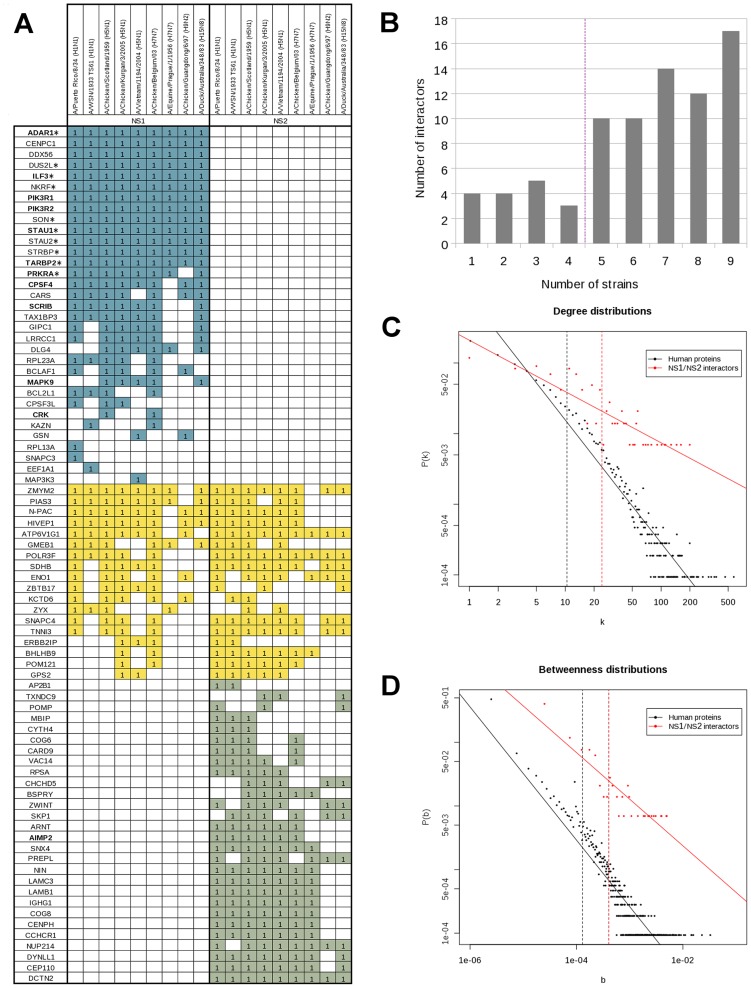
Interactions between NS1 and NS2 proteins and human host factors. (A) Yeast two-hybrid array. The 33 NS1-specific interactors are indicated in blue, 28 NS2-specific interactors in grey and shared interactors in yellow. The 11 NS1 and the single NS2 interactors described earlier are highlighted with bold letters. DRBD-containing proteins (DRBPs) are indicated with a star. (B) Frequency of interactions between individual host cell factors and NS1 and/or NS2 proteins of the 9 different influenza virus strains. (C) Degree distribution of human proteins and human proteins targeted by NS1 and/or NS2 proteins in the human interactome. P(k) is the probability of a node to connect k other nodes in the network. Solid lines represent linear regression fits. Vertical dashed lines indicate the mean degree of each distribution. (D) Betweenness distribution of human proteins and human proteins targeted by NS1 and/or NS2 proteins in the human interactome. P(b) is the probability for a node to have a betweenness value of b in the network. Solid lines represent linear regression fits. Vertical dashed lines indicate the mean betweenness value for each distribution.

To identify all cellular proteins interacting with influenza virus NS1 and/or NS2 proteins, yeast two-hybrid screens (Y2H) were carried out using NS1 and NS2 proteins from 9 different virus strains as baits (Table S1) and three cDNA libraries (from human spleen, fetal brain and respiratory epithelium). Key features of the virus strains are provided in Text S1. NS1 and NS2 proteins selected for this study are representative of the natural diversity since they are distributed all along the phylogenetic trees of known NS1 and NS2 sequences (Text S1, Figures S1 and S2 in Text S1, Alignments of NS1 and NS2 protein sequences are presented in Figures S3 and S4 in Text S1). Seventy nine non-redundant cellular proteins were identified to interact with NS1, NS2 or both and were individually retested in a pairwise array (Figure 1A). From a total of 1422 possible interactions tested (79 cellular proteins tested against 9 NS1 and 9 NS2 proteins), 562 tested positive. In this way, we identified 33 cellular proteins interacting exclusively with NS1, 28 exclusively with NS2, and 18 with both NS1 and NS2.

The vast majority (97.5%) of the NS1 and NS2 interactors are known to be expressed in the respiratory epithelium (Table S2). Twelve out of the 79 host interactors have already been reported (AIMP2, SCRIB, CPSF4, the kinases PIK3R1, PIK3R2, MAPK9, CRK and proteins with a double-stranded RNA-binding domain STAU1, PRKRA, ADAR1, TARBP2, ILF3) [9], [11], [19], [33]–[38]. 21.5% of host interactors are targeted by all virus strains (Figure 1B) and 5% appear to be strain specific. 80% of the cellular interactors bind to more than 50% of the tested NS1 and NS2 proteins indicating that the dataset is more appropriate to the identification of common rather than differential interaction profiles. Together with previously published data available in the VirHostNet database [39], we now provide a list of 111 non-redundant cellular proteins interacting exclusively with NS1, 32 exclusively with NS2 and 18 with both proteins (a complete list of influenza virus interactors is given in Table S3).

Consistent with observations from previous virus-host interactome studies, NS1 and NS2 proteins tend to interact with highly central proteins in the human interactome [40]–[43]. Indeed, the degree distribution of targeted human proteins was significantly higher than the degree distribution in the human interactome (U-test, p-value<2.2×10^−16^) (Figure 1C). Similarly, the betweenness distribution of targeted human proteins was significantly higher than the betweenness distribution in the human interactome (U-test, p-value<2.2×10^−16^) (Figure 1D). This suggests that influenza NS1 and NS2 proteins preferentially target pleiotropic cellular proteins [44].

Finally, an assessment of Gene Ontology categories revealed a significant enrichment (p-value = 3.3×10^−14^) for DRBD-containing proteins (DRBPs) in the interaction dataset. Strikingly, DRBPs were exclusively targeted by NS1 proteins. All virus strains interacted with most of the DRBPs suggesting that the direct targeting of DRBDs is of special importance for influenza A viruses.

### Impact of NS1 and NS2 cellular targets on influenza virus replication

Among the 79 NS1 and NS2 interactors identified here, 12 have been previously identified in recent genome-wide siRNA screens as modulators of viral replication - ATP6V1G1, RPL13A [21], [23], GMEB1, PIK3R2 [19], SON, EEF1A1 [23], CHCHD5, RPL23A [24] and NUP214 [22]. However, since these genome-wide siRNA screens are weakly overlapping, it is very likely that numerous modulators of viral replication have been missed or remain to be confirmed [25]–[27].

**Figure 2:**
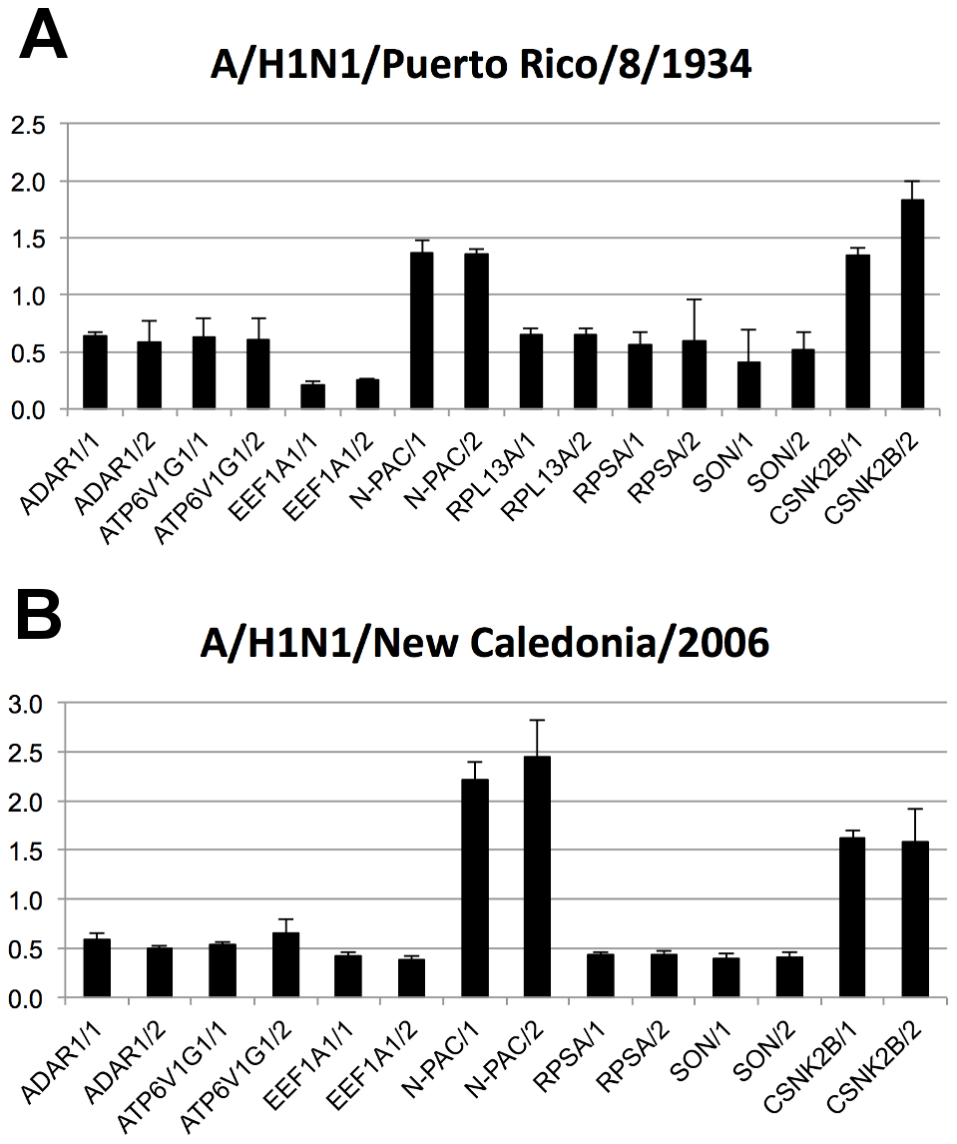
Impact of silencing of NS1 and NS2 interactors on influenza A virus replication. A549 cells were transfected with indicated siRNAs, infected at a MOI of 0.5 with A/H1N1/Puerto Rico/8/34 virus strain (A) or A/H1N1/New Caledonia/2006 virus strain (B) and the neuraminidase activity (NA) was measured in the supernatant 48 h post infection. Values are normalized to neuraminidase activity measured in supernatants of control siRNA-transfected cells and represent the mean +/− standard deviation (triplicates). ATP6V1G1 is a host dependency factor, CSNK2B is an anti-viral host factor. Both served as controls.

We have therefore performed a systematic siRNA-based screen in A549 human lung epithelial cells to explore the functional contribution of the 79 cellular NS1 and NS2 interactors to virus replication. The silencing phenotype was first tested by measuring replication of the A/H1N1/Puerto Rico/8/34 virus strain which was used in the yeast two- hybrid screen. The complete replication cycle was first probed by measuring the neuraminidase activity in the supernatant 48 h post-infection. The assay was calibrated by using siRNAs against ATP6V1G1 and CSNK2B that have been previously described as pro-viral host factor and anti-viral host factor respectively. ATP6V1G1 is a subunit of the vacuolar ATPase proton pump required for influenza A virus replication [21] while CSNK2B gene silencing increases virus replication in A549 infected cells [45]. As expected, siRNAs targeting ATP6V1G1 and CSNK2B respectively reduced and increased the neuraminidase activity in the supernatant, thus validating the assay (Figure 2A and 2B). By comparison with these controls, virus replication should be altered by at least 35% according to the threshold defined by König et al. in their genome-wide siRNA screen [22]. This threshold together with a silencing efficiency greater than 60% for each siRNAs without detectable cytotoxicity were used for a stringent selection of the pro-viral and anti-viral host factors (Table S4). These criteria are in the range applied in earlier siRNA-based screens [23], [46], [47] (information on individual silencing efficiency is also provided in Table S4).

In this way, we identified the two pro-viral host factors, ADAR1 and RPSA, and confirmed ATP6V1G1, RPL13A, EEF1A1 and SON (Figure 2A). In addition, one new anti-viral host factor, N-PAC, was identified (Figure 2A). These results were confirmed by using plaque assays, revealing a broader range of inhibition or activation of virus replication (Table S4). In conclusion, out of the 79 cellular interactors of NS1 and NS2 identified in this study, 7 were identified as possible direct modulators of A/H1N1/Puerto Rico/8/34 virus replication. Importantly, these results were confirmed with the A/H1N1/New Caledonia/2006 influenza virus strain that was not used in the yeast two-hybrid screen (Figure 2B), although RPL13A only scored positive 72 h post infection (Figure S5 in Text S1).

As it is a critical component of virus-host interaction, production of type I interferon was quantified in the supernatant of infected cells transfected with siRNAs (Table S4). Silencing of ADAR1, ATP6V1G1, BCLAF1, RPL13A and SON increased interferon production in infected cells. These proteins interact with NS1 and, except for BCLAF1, are pro-viral host factors for the virus. Therefore, 4 of the 6 pro-viral host factors are implicated in interferon production. This is consistent with the role of NS1 in interfering with the type I interferon system.

**Figure 3:**
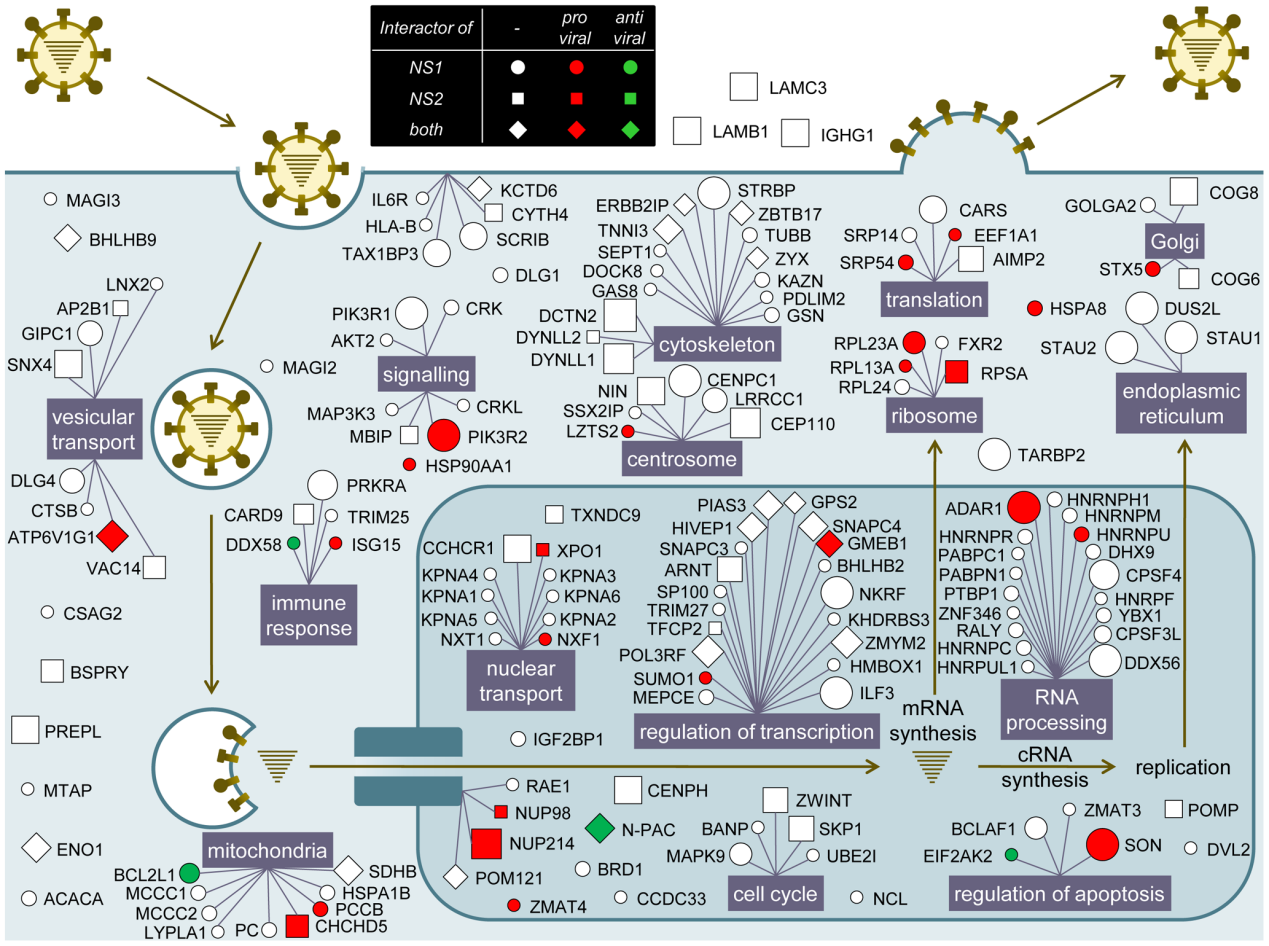
Overview of NS1 and NS2 interactors on the influenza A virus life cycle. The framework of this picture is from Brass *et al.* [21]. NS1 and NS2 interactors identified in the present study and described in the literature were placed according to information from Gene Ontology, Human Protein Atlas and HPRD. When multiple annotations were available, the most relevant for the virus was chosen. Circle node: NS1 interactor; Square node: NS2 interactor; Diamond-shaped node: NS1 and NS2 interactor. A red node is a pro-viral host factor and a green node is an anti-viral host factor according to the siRNA data from this study and genome-wide screens. The node size is proportional to the number of virus strains interacting with the host factors (interactors from the literature are often tested against a single virus strain hence appearing with a small node size).

The function and subcellular localization of cellular interactors identified in the present and published studies indicate that both NS1 and NS2 are pleiotropic proteins required for several essential steps of the viral life cycle (Figure 3). Although NS2 function in the cytoplasm remains elusive, it is shown here that NS2 mostly targets proteins of the cytoskeleton and involved in intracellular transport (Figure 3). Given that NS2 also interacts with the vRNPs, it might also mediate their transport to the plasma membrane or to the nucleus. In case of the latter, NS2 is implicated in the export of vRNPs, consistent with the observed interaction of NS2 with the NPC [13]. The targeting of transcription-regulating proteins by NS2 is much less documented. A role of NS2 in regulating influenza virus RNA genome transcription via its interaction with vRNPs has been previously proposed [15], [48]. Although such a direct interaction with the components of the vRNPs is not ruled out, our data indicate a direct targeting of the cellular transcriptional machinery by NS2 (Figure 3, box regulation of transcription). Interestingly, since NS1 also targets this process, a potential cooperation between NS1 and NS2 for the control of the cellular transcription machinery can be speculated.

NS1 proteins target several DRBPs either localized in the nucleus or in the cytoplasm [49]. These proteins are critical transcriptional or translational checkpoints. NS1 is known to inhibit the activation of PKR, one of the major interferon-inducible antiviral effectors, through direct interaction [6]. More recently, SON has been described to be important for the trafficking of influenza virions [23]. Here, we confirmed that SON is essential for viral replication and suggest that this activity could be related to the NS1 protein. ADAR1 and PKR have an opposite effect on virus replication although they are both induced by type I interferon.

### ADAR1 is a pro-viral host factor for influenza A virus replication

**Figure 4:**
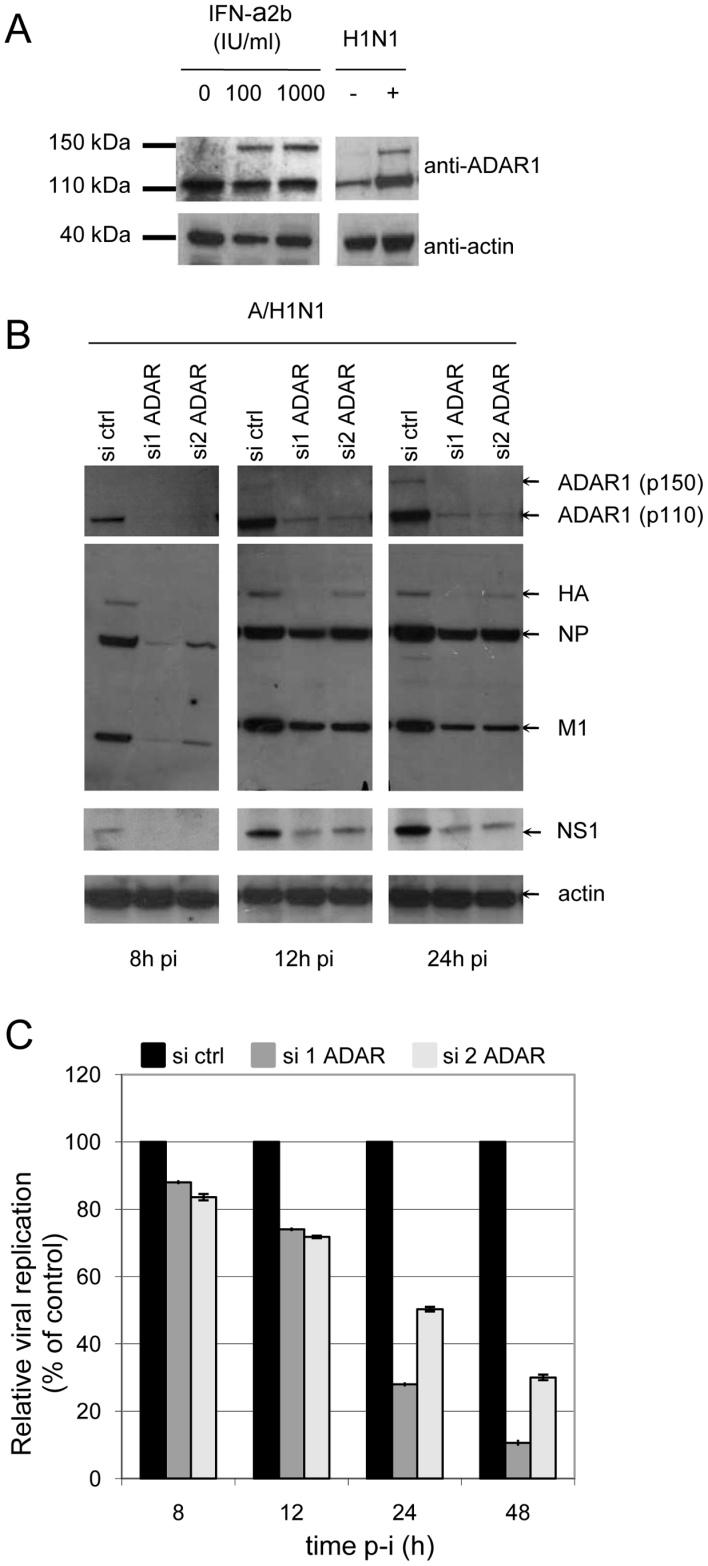
ADAR1 is a pro-viral host factor for influenza A virus replication. (A) ADAR1 expression in A549 cells upon interferon treatment or H1N1 infection. A549 cells were incubated with indicated concentration of interferon-(IFN)-α2b or infected with influenza A virus and analysed 24 h later by western blot for expression of ADAR1 and actin that served as loading control. (B) Silencing of ADAR1 impairs viral protein expression. A549 cells were transfected with negative control siRNA (Ctrl) or two distinct siRNA targeting ADAR1 for 48 h and infected with A/H1N1/New Caledonia/2006 virus strain. ADAR1 and viral protein expression were assessed in cell lysates by western blot at indicated time points. (C) Silencing of ADAR1 reduces virus titers. Determination of NA in supernatants 8 h, 12 h, 24 h and 48 h post infection. Values are normalized to cells transfected with control siRNA.

ADAR1 is a type I interferon-induced protein that is expressed in human lung [29], [30] and interacts with all tested NS1 proteins. The 150 kDa interferon-inducible ADAR1 isoform is expressed in A549 cells upon influenza A virus infection and by type I interferon. The constitutive 110 kDa ADAR1 isoform was only induced upon infection indicating that ADAR1 expression can also be controlled by an interferon-independent mechanism, at least in the setting of an influenza A virus infection (Figure 4A). ADAR1-specific siRNAs efficiently reduced the expression of ADAR1 isoforms and blocked their induction upon infection (Figure 4B). The silencing of ADAR1 inhibited virus release from 15% at 8 h post-infection to 90% at 48 h post infection (Figure 4C). Expression of viral proteins (here HA, NP, M1 and NS1) was also significantly reduced as early as 8 h post infection. NS1, NP and M1 expression was delayed while HA expression remained very low until 24 h post infection (Figure 4B). Thus, ADAR1 is a pro-viral host factor for virus protein expression and virus production.

### Mapping of the ADAR1-NS1 interaction sites

**Figure 5:**
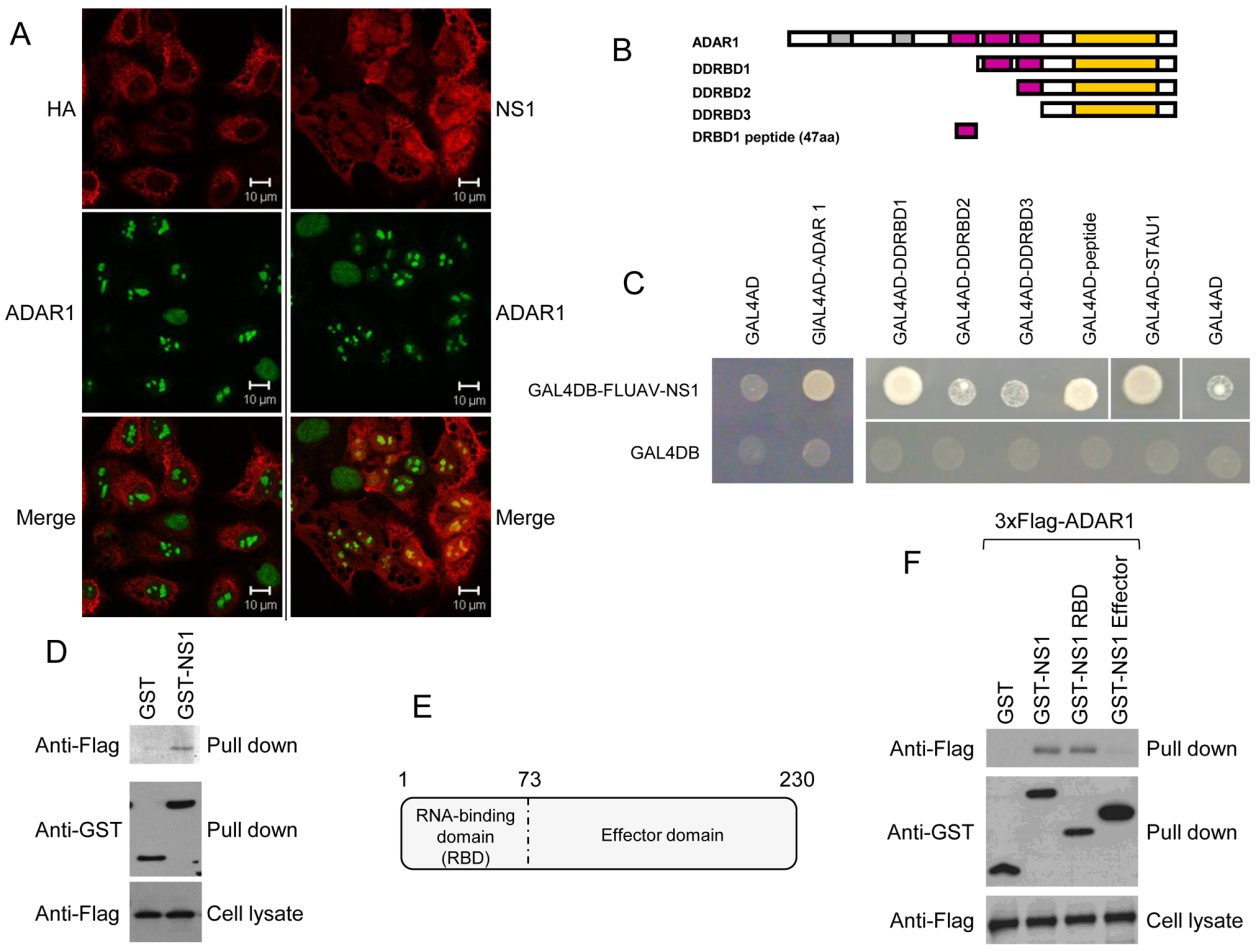
Mapping of ADAR1-NS1 interaction sites. (A) Subcellular localization of ADAR1 (green), HA and NS1 (red) 8 h post infection of A549 cells. (B) Schematic representation of full-length and truncated ADAR1 proteins. Zα DNA-binding domain (grey boxes), dsRNA binding domain (DRBD, purple boxes), adenosine deaminase domain (yellow boxes). (C) ADAR1 interaction mapping. Yeast diploids co-expressing full length A/H1N1/Puerto Rico/8/34 virus NS1 protein fused to the Gal4 DNA-binding domain and full-length ADAR1 or ADAR1 truncated mutants fused to Gal4 activating domain were spotted onto a plate containing medium without histidine. STAU1 is a positive control for interaction with NS1 [10]. Controls with empty vectors (Gal4-AD and Gal4DB) show no auto-activation induced by the different constructs. (D) GST pull-downs were performed with GST alone or GST fused to NS1 after co-expression of a 3×Flag-tagged DRBD1 peptide in HEK293T cells and incubation of cell lysates with glutathione-Sepharose beads. Cell lysates and pull-downs were analyzed by western blot using antibodies against GST or 3×Flag. (E) Schematic representation of the NS1 protein with its two domains: the N-terminal RNA-binding domain (RBD, 1-73) and the effector domain (74-230). (F.) NS1 interaction mapping. GST alone or GST fused to NS1, NS1 RBD or NS1 effector were co-expressed with 3×Flag-tagged ADAR1 in HEK293T cells and their interaction was assessed after co-affinity purification with glutathione sepharose beads and immunoblotting.

Immunofluorescence revealed that ADAR1 is diffusely distributed in the nucleus and relocalized in nuclear structures in influenza virus-infected cells (Figure 5A). In these structures ADAR1 colocalized with NS1 but not with HA for which no interaction with ADAR1 could be detected. As NS1 interacts with several DRBD-containing proteins, the NS1 binding site in ADAR1 could be a DRBD. Amino acid sequence alignment of DRBDs revealed a conserved region of 47 amino acid residues within the two firsts DRBD of ADAR1 (Figure S6 in Text S1). A set of 4 ADAR1 deletion mutants, differing in their number of DRBDs, and a plasmid encoding the 47 amino acid residues of the first DRBD were constructed (Figure 5B). In a yeast two-hybrid array, ADAR1 interacted with NS1 even in the absence of its first DRBD while interaction was completely abrogated when the first two DRBDs were deleted. The peptide of 47 amino acid residues also interacted with NS1 (Figure 5C) in the array and in GST pull-down assays (Figure 5D). Thus, ADAR1 displays two potential NS1 interaction sites located on the first two double-stranded RNA-binding domains.

To validate these results the NS1 RNA-binding domain (RBD) and effector domain fused to GST were used in pull-down experiments for the mapping of NS1 interaction with 3×Flag tagged ADAR1 after co-expression in HEK293T cells (Figure 5E). Full-length NS1 and NS1 RBD domain efficiently co-precipitated ADAR1 but not the effector domain (Figure 5F) indicating that NS1 interacts with ADAR1 through its RBD. GST pull-down and RNAse A treatment showed that RNA is marginally involved in the NS1-ADAR-1 interaction (Figure S7 in Text S1). A mutant of NS1 that lacks double-stranded RNA-binding activity still interacts with ADAR1, albeit with reduced efficiency (Figure S8 in Text S1) confirming that RNA is not strictly required for NS1 interaction with ADAR1.

### NS1 and influenza virus infection enhance ADAR1 editing activity

**Figure 6:**
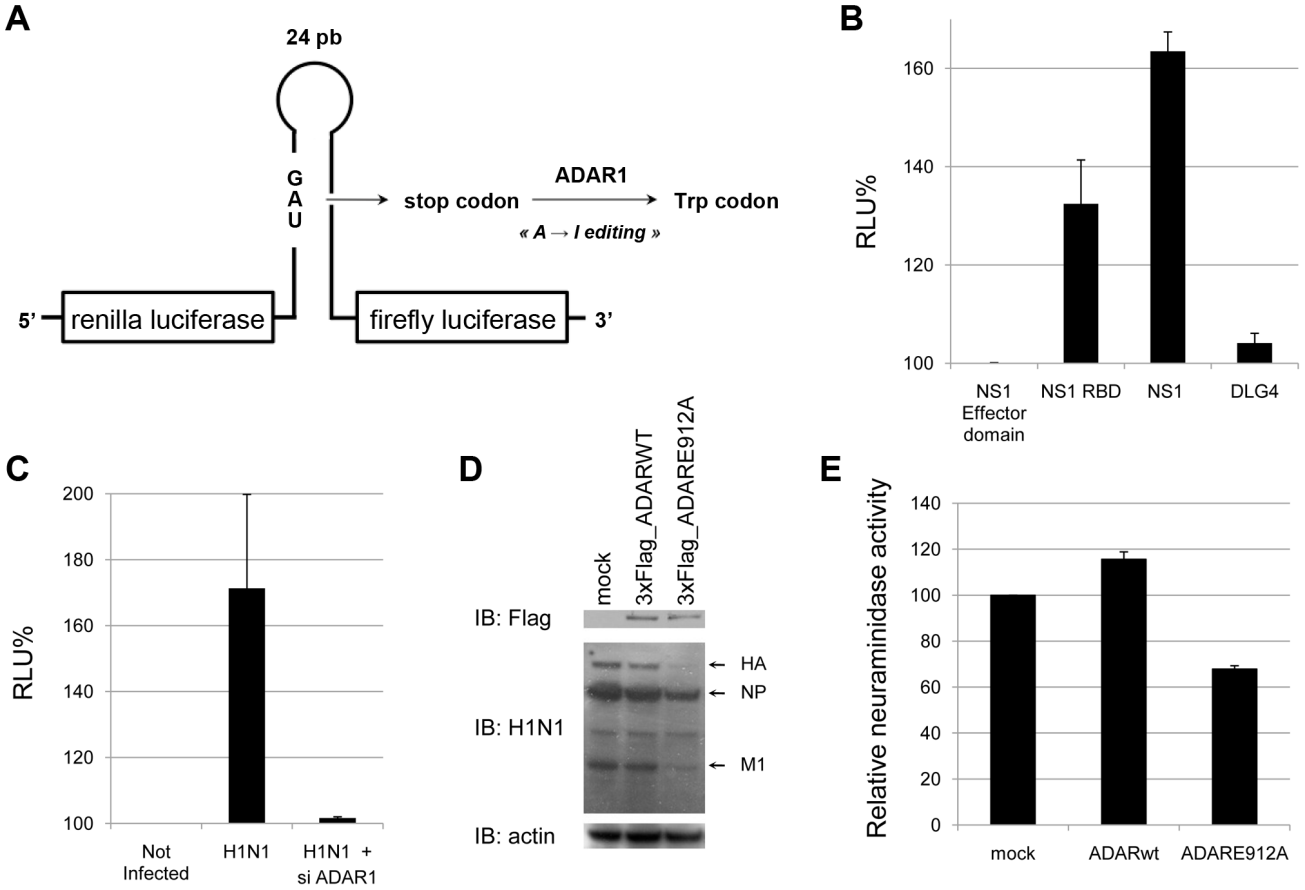
Enhancement of ADAR1 editing activity by influenza virus NS1. (A) The RNA editing reporter system is composed of the hepatitis D virus minimal sequence edited by ADAR1 and positioned in-between the Renilla luciferase and the Firefly luciferase coding sequence, respectively. The unedited reporter has a stop codon that is converted into Trp codon upon A to I editing by ADAR. Hence, editing is correlated with Firefly luciferase expression while Renilla luciferase expression is used as an internal control. (B) HEK293T were co-transfected with the editing reporter, ADAR1 and NS1 (full-length, RNA-binding domain or effector domain) or the control protein DLG4. Two days post transfection, luciferase activities were determined by luminescence measurement. Data are expressed as percentage of the luciferase activity detected in cells expressing the NS1 effector domain (relative light unit, RLU). (C) Editing activity in HEK293T expressing or not ADAR1, transfected with the editing reporter and infected with influenza virus H1N1. (D, E) A549 cells were transfected with plasmids encoding for wild type or catalytically inactive ADAR1 (ADAR1 E912A, Text S1). Forty eight hours later, cells were infected with the A/H1N1/New Caledonia/2006 virus strain at a MOI of 0.5. After an additional 48 h incubation period, expression of ADAR1 and viral proteins was assessed in cell lysates by using western blot (D) and neuraminidase activities were measured in supernatants (E). Values are normalized to mock-transfected cells.

To evaluate the functional impact of ADAR1-NS1 interaction on the catalytic activity of the enzyme, an original editing reporter system was constructed. This reporter system consists of a 24 nucleotide-long minimal ADAR1 substrate derived from the sequence of the antigenome of the hepatitis delta virus that is edited by this enzyme [50]. In this sequence, ADAR1 editing activity changes a stop codon into a tryptophane codon (Figure 6A) [51]. The reporter plasmid contains the ADAR1 substrate sequence inserted in frame in-between the Renilla and the Firefly luciferase genes (Figure 6A). In this configuration, the Firefly luciferase activity reflects the extend of editing and thus ADAR1 activity, leading to the conversion of the stop codon into the tryptophane codon. ADAR1 was co-expressed in HEK293T cells with NS1 or its RBD and with the editing reporter construct. The NS1 effector domain or DLG4, which does not bind to ADAR1 (data not shown), was used as negative control in analogous co-transfection experiments. NS1 RBD and full-length NS1 increased the Firefly luciferase signal by 30% and 60% respectively (Figure 6B) suggesting that NS1 can cooperatively interact with ADAR1 via its RNA-binding domain to promote ADAR1 editing activity (Figure 6B). Editing activity was also analyzed in the context of influenza virus infection after expression of the editing reporter construct (Figure 6C). H1N1 influenza virus infection increased the editing activity of ADAR1 by 70% and this was completely reversed when ADAR1 expression was silenced by RNA interference.

To validate these observations, a catalytically inactive ADAR1 (E912A) mutant was constructed [52]. Unfortunately, A549 cells became refractory to plasmid DNA transfection after siRNA transfection, thus precluding functional tests of the mutant in this cell line (not shown). As an alternative, we tested a potential transdominant negative effect of the ADAR1 mutant on influenza virus growth. The catalytically inactive ADAR1 (E912A) mutant construct was therefore transfected into A549 cells and virus growth in these cells was compared to the one achieved with mock-transfected cells or in wild type ADAR1-transfected cells. Viral protein expression was reduced in A549 cells expressing the ADAR1 mutant compared to control cells (Figure 6D). Consistent with this result, neuraminidase activity in the supernatant was also significantly reduced (Figure 6E). Importantly, since influenza A virus infection induces endogenous ADAR1 expression, the impact of the ADAR1 mutant is most likely underestimated in this experimental system. We therefore concluded that the RNA editing function is required for the pro-viral activity of ADAR1.

### Dengue virus NS3 cooperatively interacts with ADAR1

**Figure 7:**
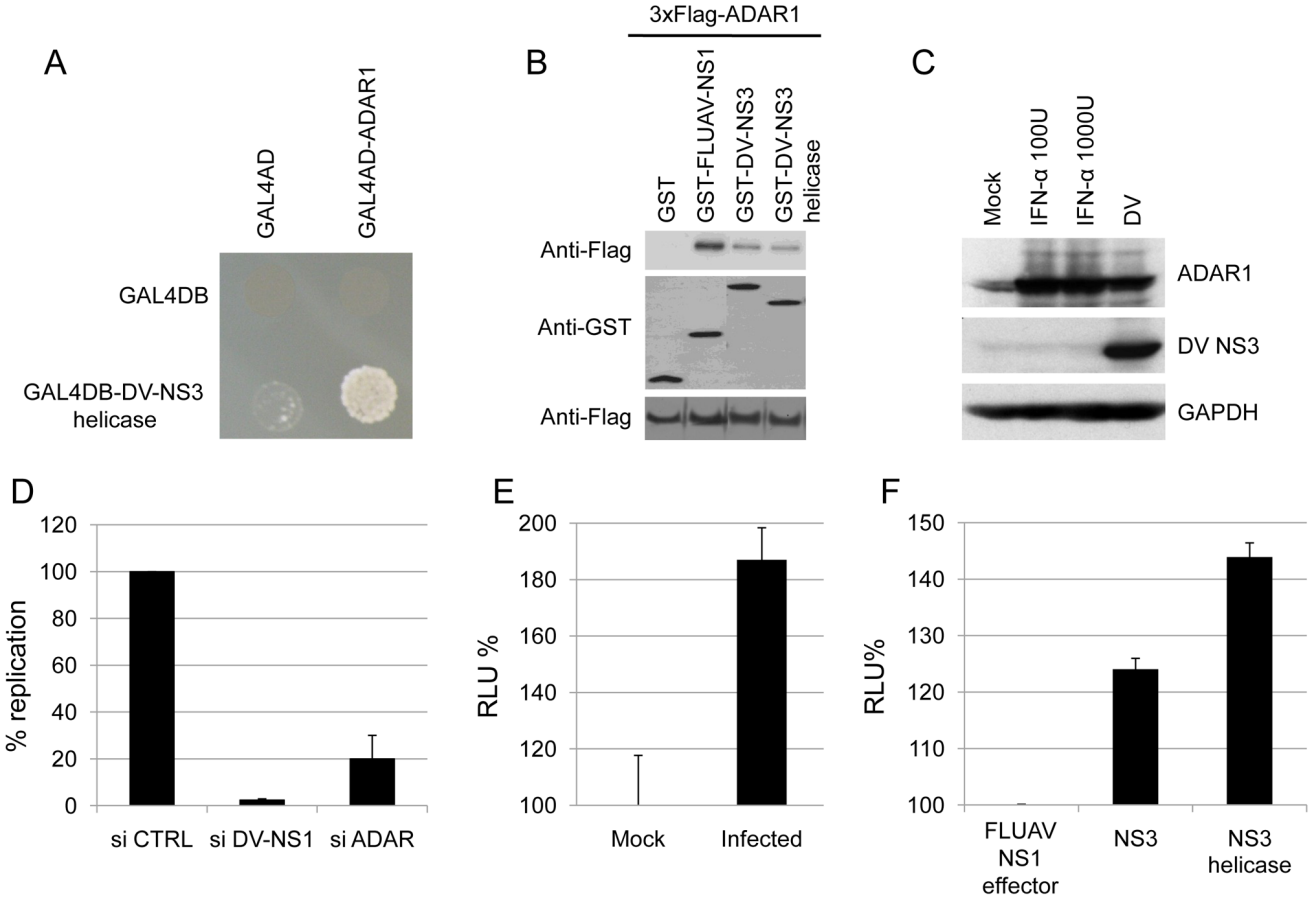
Dengue virus NS3 protein also interacts with ADAR1. (A) Pairwise Yeast diploids co-expressing dengue virus type 2 NS3 helicase fused to Gal4 DNA-binding domain and ADAR1 fused to Gal4 transactivation domain were plated onto a selective medium lacking histidine to determine interaction-dependent transactivation of the HIS3 reporter gene. Negative controls are vectors without insert (pPC97 and pPC86 for bait and prey respectively). (B) HEK293T cells were co-transfected with 3×Flag-tagged ADAR1 and GST-tagged full-length NS3 of dengue virus (GST-DV-NS3) or its helicase domain (GST-DV-NS3 helicase) or GST-tagged full-length NS1 of influenza virus (GST-FLUAV-NS1) as a positive control. Proteins bound to glutathione sepharose beads were analyzed by western blot using antibodies against GST or 3×Flag. (C) ADAR1 expression in Huh-7 cells upon interferon treatment or dengue virus infection. Huh-7 cells were incubated with 0, 100 or 1000 IU/ml of interferon- (IFN)-α2b or infected with dengue virus and analyzed 24 h later for expression of ADAR1, NS3 and GAPDH. (D) Impact of ADAR1 silencing on dengue virus replication in Huh-7. Data are expressed as the percentage of virus titer obtained with control siRNA-transfected cells. A siRNA targeting dengue virus NS1-coding region (siDV-NS1) was used as positive control for the silencing. (E) ADAR editing activity in Huh-7 cells infected with dengue virus. (F) Dengue virus NS3 contribution to ADAR1 editing activity. The NS1 effector domain of influenza virus, full-length NS3 of dengue virus or its helicase domain were co-expressed with ADAR1 and the editing reporter in HEK293T cells. Luminescence reflecting Firefly and Renilla luciferase activities was measured 48 h post-transfection. The influenza virus NS1 effector domain does not interact with ADAR1 and was used as negative control. Data are normalized to the values obtained with the NS1 effector domain. RLU, relative light unit.

During the course of a systematic screening for virus-host protein-protein interactions with a yeast two-hybrid system, we also identified ADAR1 as an interactant of the NS3 protein of dengue virus type 2. This interaction was confirmed in a yeast two-hybrid pairwise array (Figure 7A). As for NS1 of influenza A virus, interaction between NS3 and ADAR1 was validated by GST pull-down experiments (Figure 7B) and also in this case, RNA contributed to this interaction only to a very minor extent (Figure S9 in Text S1). Both ADAR1 isoforms were induced upon dengue virus infection as well as upon type I interferon treatment of Huh-7 cells (Figure 7C). Silencing of ADAR1 expression by RNA interference (Figure S10 in Text S1) resulted in a strong decrease of dengue virus replication (Figure 7D). This result was confirmed with a subgenomic dengue virus replicon stably replicating in Huh-7 cells, indicating that ADAR1 acts at a post-entry step in the dengue virus life cycle (Figure S11 in Text S1). Likewise, as observed for influenza virus, dengue virus infection strongly increased the editing activity of ADAR1 (Figure 7E). In fact, full-length NS3 and the helicase domain increased the Firefly signal by 24% and 44% respectively, suggesting that NS3 cooperatively interacts with ADAR1 to enhance its editing activity (Figure 7F).

In conclusion, both influenza virus and dengue virus (i) induce over-expression of ADAR1, (ii) interact with ADAR1 through the RNA-binding domain of influenza virus NS1 and the helicase domain of dengue virus NS3, (iii) enhance the editing activity of ADAR1 and (iv) are dependent on ADAR1 expression for efficient virus replication.

## Discussion

This study describes an exhaustive interaction profile for NS1 and NS2 proteins of 9 influenza virus strains. More than 560 interactions between 79 cellular proteins and NS1 and NS2 were identified. Thirty-three cellular proteins interacted exclusively with NS1, 28 exclusively with NS2, and 18 with both NS1 and NS2. Since NS1 and NS2 are the products of alternatively spliced RNAs, shared interactions may reflect binding to the common N-terminal 10 amino-acid residues long sequence. This result suggests that influenza viruses have evolved two proteins to interact with cellular proteins that are potentially essential for them.

Twelve out of the 79 NS1 and NS2 cellular interactors have already been reported in the literature, demonstrating the reliability and robustness of our screening approach. For NS1, there is a strong overlap with hits published by others (11 of the 51 interactors identified in the present study, which is well above the average overlap) [41], [53], suggesting that the NS1 interactome dataset is now close to completion. In case of NS2, only 4 cellular interactors have been published and one of them, AIMP2, has been confirmed in our screens. Although 46 new NS2 interactors have been identified, it is difficult at this stage to estimate the completion level of the NS2 interactome due to the lack of published interaction data.

Overall, most of the cellular targets interacted with the majority of NS1 or NS2 proteins of the different influenza viruses arguing that we have identified highly relevant and evolutionary conserved interactions. Interestingly, a significant proportion of these proteins is also targeted by other viruses (44.7%, exact Fisher test, p-value<2.2×10^−16^) indicating that these cellular proteins are likely to be involved in a generic process of viral infection [39].

Our interaction dataset indicates that NS1 and NS2 proteins are likely to be involved in multiple steps of the viral replication cycle, paving the way for challenging functional explorations. This was largely unexpected for NS2, which is known to be involved in the nuclear export of the vRNPs. Its interaction with the cytoskeleton appears particularly interesting for further studies. Although the pleiotropic nature of NS1 is well established [54], our study provides new insights into the breadth of interactions and activities of this regulatory protein. In addition to the 67 new interactors, the current dataset also provides additional information on previously known interactors and related targeted functions. For instance, the CPSF4 interaction with NS1 has been described as a potential therapeutic target [55] and is confirmed in our study. Three NS1 proteins also interacted with CPSF3L, a protein participating in the endonuclease activity of CPSF, suggesting that the corresponding viruses evolved alternative strategies to interfere with the cellular 3′end mRNA processing [56].

The phenotypic analysis of the cellular targets of NS1 and NS2 by RNA interference revealed an enrichment in modulators of influenza virus replication, further validating the interaction dataset. Indeed, out of the 79 cellular interactors of NS1 and NS2 identified in this study, 7 revealed to control positively or negatively the replication of two influenza virus strains. Interaction profiles suggest that the data could be extrapolated to other strains with the noticeable exception of RPL13A, an exclusive target of A/H1N1/Puerto Rico/8/34 NS1. The validation rate of cellular interactors by RNAi reached about 9% (15.2% when data from the literature are included) and is similar to that of Shapira *et al.* [19] while the validation rate of virus replication modulators identified from genome-wide siRNA screens ranges from 0.75 to 1.5%. Therefore, combining interactomic screens with genetic screens drastically enhances the rate of functional validation, providing lists of cellular proteins strongly enriched in pro- and anti-viral host factors (exact Fisher test, p-value = <2.1 10^−4^, Text S1).

Interaction of NS1 with some members of the DRBD protein family have been sporadically documented [6], [10], [19]. Here we observed a massive enrichment of the DRBD protein family in our NS1 interactome for which we used 9 different influenza virus strains. One hundred and sixty five independent screens have been performed with other viral baits using the same cDNA libraries (45 with the fetal brain cDNA library, 31 with the respiratory epithelium library and 89 with the spleen library). The GO term “Double-stranded RNA-binding domain (DRBD) containing proteins” has never been enriched in any of these screens while it was enriched for the 9 tested influenza virus strains. Reciprocally, a large diversity of other GO terms was enriched in these different screens and in screens performed by other laboratories using the same libraries. Therefore, we could be confident that the DRBD containing proteins enrichment reflects a real propensity of NS1 to interact with this protein family. This is most likely reflecting the ability of NS1 to interact with the double-stranded RNA-binding domain of cellular partners through its own RNA-binding domain. Two DRBD-containing proteins, SON and ADAR1, were found to be essential for virus replication. Conflicting results on the role of ADAR1 for virus replication have been published. Initially suspected to have an antiviral activity because of its induction by interferon, ADAR1 appears to promote the replication of several viruses (measles virus, vesicular stomatitis virus, hepatitis delta virus, human immunodeficiency virus type 1 and Kaposi's sarcoma-associated virus). In contrast ADAR1 was reported to display an antiviral activity against hepatitis C virus and lymphocytic choriomeningitis virus [28], [57]. Concerning influenza A virus, two studies provided evidence for an antiviral role of ADAR1. Mice lacking IKKε become highly susceptible to influenza virus infection, express ADAR1 only to low amounts and show a reduced editing of matrix M1 mRNA isolated from infected lung. However, since IKKε knock-out also strongly affects the expression of other type I interferon-stimulated genes, the susceptibility of these mice to infection could not be attributed to a unique defect in ADAR1 activity [58]. An increased cytopathic effect of influenza A virus has been observed in mouse cells derived from non-viable embryos unable to express the p150 isoform of ADAR1. However, this effect was not correlated to an increased virus replication [59]. In the present study, we show that inhibiting ADAR1 expression by RNA interference reduced viral protein expression and drastically impaired virus replication. Thus, ADAR1 appeared as an important host dependency factor for influenza viruses.

Several studies have demonstrated a role of ADAR1 in modulating interferon signaling. Inducible ADAR1 disruption in mice causes a global interferon response [31]. Mutations in ADAR1 responsible for Aicardi-Goutières syndrome in humans are associated with upregulation of interferon-stimulated genes [60]. ADAR1 also suppresses measles virus-induced production of interferon-β mRNA [61]. Here, we show that interferon-β is enhanced in ADAR1-deficient cells after infection with influenza A virus. NS1 is a well-known antagonist of the antiviral response. Its mode of action is pleiotropic including interference with signaling induced by RIG-I like receptors (RLRs) [62]. A combined action of ADAR1 and NS1 protein is suggested by our results. The double-strand RNA editing activity of ADAR1 produces double-strand RNA with I:U pairs instead of A:U pairs. Interestingly, I:U-containing double-strand RNA can suppress the induction of interferon-stimulated genes [63]. Conceivably NS1 might potentiate the hyperediting of an as yet unknown double-strand RNA substrate and thus interfere with interferon induction. A similar mechanism can be expected for dengue virus NS3 protein. Both NS1 and ADAR1 also interfere with PKR activity [6], [64]. ADAR1 and PKR are recognized by non overlapping domains of NS1 (respectively the RNA binding domain and the effector domain [7]). Thus, both NS1 and ADAR1 could sequester double-strand RNA or could form inactive complexes, suppressing PKR-mediated proapoptotic and interferon-mediated amplification activities.

Influenza A NS1 protein is considered as a valid target for the development of antiviral drugs. The druggability of NS1 has been demonstrated in a proof-of-concept study with an inhibitory peptide derived from CPSF30, a cellular protein that interacts and interferes with the effector domain of NS1 [55]. Such a strategy can be extended to other NS1 interactors once the interacting sequences have been mapped and the 3D structure is solved. The interacting sequences, *e.g.* the ADAR1-derived 47 amino acid peptide, could then be used for the design of low molecular weight compounds. In this respect, the systematic screening for protein-protein interactions between a virus and its host cell identifies cellular proteins promoting or restricting virus replication. Interference with these interactions may offer new alternatives to enlarge the diversity of potential therapeutic targets and prevent the emergence of resistance caused by rapid viral adaptation. Small molecules targeting these host interaction surfaces and developed for other therapeutic purposes could now be tested for their ability to control virus replication. Concerning ADAR1, new inhibitors of the RNA editing activity are being screened and could be tested for their capacity to block the replication of influenza A virus or anti-dengue virus [65]–[67]. The dual luciferase editing reporter described in this study is well suited for screening RNA editing inhibitors at a high throughput level.

## Materials and Methods

### Cells

Human HEK293T and human lung adenocarcinoma A549 cells were maintained in Dulbecco's modified Eagle's medium (DMEM) supplemented with 10% heat-inactivated fetal bovine serum (FBS), 50 IU/ml penicillin G, 50 µg/ml streptomycin, at 37°C under 5% CO_2_. Huh-7 cells were grown in DMEM supplemented with 2 mM L-glutamine, non-essential amino acids, 100 IU/ml of penicillin, 100 µg/ml of streptomycin and 10% fetal calf serum.

### Yeast two-hybrid

Influenza ORFs (Text S1) were transferred from pDONR207 into bait vector (pPC97, Lifetechnologies) to be expressed as Gal4-DB fusions in yeast. Bait vectors were transformed into AH109 (bait strain, Clontech [68]), and human spleen, fetal brain and respiratory epithelium Gal4-AD-cDNA libraries (each containing more than 10^6^ primary clones) were transformed into Y187 (prey strain, Clontech). Single bait strains were mated with prey strains and diploids were plated on SD-W-L-H+ 10 mM 3-AT medium. Each screen has covered more than one time the libraries. Positive clones were maintained onto this selective medium for 15 days to eliminate any contaminant AD-cDNA plasmid. AD-cDNAs were PCR-amplified, sequenced and analyzed using pISTil [69]. Cellular ORFs (interacting domains found in Y2H screens) were amplified from a pool of human cDNA libraries or from a plasmid encoding the corresponding cDNA from the MGC collection (IMAGE consortium) using KOD polymerase (Toyobo) and cloned by recombinational cloning into pDONR207 (Invitrogen). Primers contained the attB1.1 and attB2.1 gateway recombination sites. All entry clones were sequence-verified and individually transferred by recombinational cloning into a prey vector (pPC86, Invitrogen) to be expressed as Gal4-AD (activating domain) fusion in yeast. Pairwise yeast two-hybrid interaction analyses were also performed by yeast mating using Y187 and AH109 yeasts strains (Clontech [68]), as described in [70]. Bait and prey strains were mated in an all-against-all array (together with negative controls, either empty bait vector or empty prey vector) and plated on a selective medium lacking histidine and supplemented with increasing concentrations of 3-AT (0, 5, 10, 15 mM) to test the interaction-dependent transactivation of HIS3 reporter gene. Interactions were scored as positive if observed in at least 2 out of 3 independent arrays. When yeasts containing an empty bait vector and a prey vector were still able to grow, the corresponding proteins were rejected as being auto-activators and thus false positives.

### Topological analysis

The R statistical environment was used to perform statistical analysis and the igraph R package to compute network topology measures [71].

Protein-protein interaction networks are formed by a set of N nodes (or vertices) representing proteins connected by E edges representing physical interactions between these proteins. The topology of protein-protein interaction networks can be described by a set of measures:

The degree or connectivity (k) of a node v in a graph is a local centrality measure which summarizes the number of edges that are incident to this node v.

The betweenness (b) of a node v in a graph is a global centrality measure which can be defined by the number of shortest paths going through this node v and is normalized by twice the total number of protein pairs in the graph 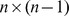. The equation used to compute betweenness centrality, b(v), for a node v is:
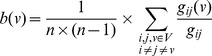
where g_ij_ is the number of shortest paths going from node i to j, i and j ∈ V and g_ij_(v) the number of shortest paths from i to j that pass through the node v.

### Functional analysis

DAVID database was used for functional annotation [72]. DAVID functional annotation chart tool was used to perform Gene Ontology categories analysis. Gene Ontology terms with a Benjamini-Hochberg corrected p-value smaller than 5.10^2^ were considered as significantly overrepresented.

### siRNA screening

5 pmoles of each siRNA (stealth select RNAi, Invitrogen) were arrayed in 96 plates in 10 µl of OptiMEM (2 siRNAs per gene). After 20 minutes of room temperature incubation with a transfection agent (0.2 µl of lipofectamine RNAiMAX in 10 µl of OptiMEM), siRNA-transfection agent mix was added to 3.10^4^ A549 suspension cells. Cells were incubated for 48 hours at 37°C and 5% CO_2_ before influenza A virus infection at MOI 0.5. At indicated time post-infection, supernatants were titered.

### Virus infection

siRNA-transfected cells were washed twice with DMEM and infected with the A/H1N1/Puerto Rico/8/34 strain or the A/H1N1/New Caledonia/2006 strain at indicated MOI in infection medium (DMEM supplemented with 0.2 µg.ml^−1^ TPCK-trypsin (Sigma)). After 1 h at 37°C, the inoculum was discarded and cells were washed again and incubated in infection medium at 37°C and 5% CO_2_.

### Neuraminidase assay

Standard fluorimetric assay was used to measure influenza virus neuraminidase activity [73]. Influenza virus neuraminidase is able to cleave the methyl-umbelliferyl-N-acetylneuraminic acid (4-MUNANA, Sigma) yielding a fluorescent product that can be quantified. In 96-black plate, 25 µl infection supernatants were diluted in 25 µl D-PBS containing calcium and magnesium and the reaction was started with 50 µl of 20 µM 4-MUNANA. After 1 h incubation at 37°C, the reaction was terminated by adding 100 µl of glycine 0.1 M, 25% ethanol pH 10.7. Fluorescence was recorded with TECAN infinite M1000 instrument at 365 nm excitation and 450 nm emission wavelengths.

### Co-affinity purification, RNase A digestion and immunoblotting

ADAR1 was transferred from pDONR207 to pCIneo3×Flag (kind gift of Dr Y. Jacob, Pasteur Institute, Paris, France). NS1 and NS3 constructs were transferred in pDEST27 (Invitrogen). Plasmids coding for mutant NS1 (pCAGGS-NS1-R38AK41A) and control NS1 (pCAGGS-NS1) are kind gifts from A. Garcia-Sastre (Mount Sinai School of Medicine, New York). HEK293T cells were transfected in 6-well plates using JetPEI transfection reagent (Polyplus Transfection). 48 h post-transfection, cells were lysed in a cold extract buffer (20 mM Tris-HCl pH 8.0, 150 mM NaCl, 1 mM EDTA, 0.5% Igepal and a protease inhibitor cocktail (Roche)). Protein extracts (300 µg) were incubated overnight with Glutathione Sepharose 4B beads (GE Healthcare) at 4°C. Beads were then extensively washed with the cold extract buffer, proteins were separated by SDS-PAGE and transferred to a nitrocellulose membrane. GST-tagged viral proteins and 3×FLAG-tagged cellular proteins were detected using standard immunoblotting techniques with a mouse peroxidase-conjugated anti-GST monoclonal antibody (Sigma) or a mouse peroxidase-conjugated anti-FLAG M2 monoclonal antibody (Sigma). When indicated, pull-downs were treated with 2 µg of RNAse A (Invitrogen) in a buffer containing 100 mM NaCl for 30 min at 4°C. Proteins bound and released in the supernatants were then detected by immunoblotting using anti-ADAR (Sigma), anti-influenza A virus (Chemicon) and anti-NS1 antibodies (Abcam). Anti-actin antibody was purchased from Sigma.

### Editing assay in transfected cells

HEK293T were transfected in 24-well plates with a total of 1 µg plasmid DNAs (editing reporter plasmid, 3XF-ADAR1 and plasmids coding for indicated viral proteins) using the JetPEI. 24 h post-transfection, cells were seeded in 96-well plates in DMEM and incubated for 24 h. The Dual-Glo Luciferase Assay System (Promega) was then added to measure both Firefly and Renilla luminescence activities using the TECAN infinite M1000 instrument. Relative Light Unit (RLU) is the ratio of luminescence from FLUC to luminescence from RLUC.

### Editing assay in infected cells

For influenza virus, HEK293T cells were seeded at 20,000 cells/well in 96-well plate and were transfected or not with anti-ADAR1 or control siRNAs, 24 h prior transfection with the editing reporter plasmid. 24 h post transfection, cells were infected influenza A virus at MOI 10 in DMEM supplemented with 10% FCS. 24 h post-infection cells were subjected to the procedure of editing assay described above. For dengue virus, Huh-7 cells were seeded at 3.10^5^ cell/well in 12-well plates and were transfected or not with anti-ADAR1 or control siRNAs, 24 h day prior transfection with the editing reporter. 24 h later, cells were infected with dengue virus type 2 with an MOI of 20. Luciferase values were measured as described above.

### Indirect immunofluorescence microscopy

A549 cells were infected with influenza A virus at MOI 3 in DMEM supplemented with 50 IU/ml penicillin, 50 µg/ml streptomycin and 0.25 µg/ml TPCK-trypsin. Eight hours post-infection cells were fixed with 4% formaldehyde for 30 min and permeabilized with 0.5% Triton X100. Double staining were performed by incubation with mouse monoclonal antibodies anti-NS1 (clone 1A7, kindly provided by Robert G. Webster) or anti-HA (Abcam) and rabbit anti-ADAR (Sigma) in combination with Alexa 488-labeled anti-rabbit F(ab)′2 fragment and Alexa 546-labeled anti-mouse F(ab)′2 fragment (Molecular Probes). Analyzes were performed with a laser-scanning confocal microscope (Axioplan LSM510 v3.2 (Zeiss)) and images were processed using LSM Image Browser (Zeiss).

## Supporting Information

Table S1 **Origin of NS1 and NS2 proteins used in this study.** Sequences are available through ViralORFeome database, using indicated clone identification number.
(XLS)

Table S2 **NS1 and NS2 interactors expressed in lung and trachea.**
(XLS)

Table S3 **Complete list of NS1 and NS2 interactors.** Y2H: interactors identified in this study by yeast two-hybrid screening and arrays. Literature: interactors mined by text mining.
(XLS)

Table S4 **List of host proteins identified in Y2H screens and their impact on virus replication and type I interferon β production.** Data are normalized to controls. NT: not tested. ND: not detectable. The control value in this experiment is 2 pg/ml. The detection limit corresponds to 1.2 pg/ml. Therefore we cannot conclude that a gene is a positive regulator when its silencing leads to a not-detectable level of IFN.
(XLS)

Text S1 **includes supporting methods as well as key features of the chosen virus isolates, NS1 and NS2 protein sequences analyses (Figure S1 to Figure S4), identification of additional host factors 72 h post infection (Figure S5), molecular characterization of ADAR1 interaction with influenza NS1 protein and with dengue virus NS3 protein (Figure S6 to Figure S9), additional functional data in Huh-7 cells (Figure S10 and Figure S11).**
(DOC)
