# Supplementary material for: The Interactomes of Influenza Virus NS1 and NS2 Proteins Identify New Host Factors and Provide Insights for ADAR1 Playing a Supportive Role in Virus Replication
Source: PLoS Pathog. 2013 Jul 4;9(7):e1003440. doi: 10.1371/journal.ppat.1003440 (PMC3701712; doi:10.1371/journal.ppat.1003440)
Supplement: Text S1 — includes supporting methods as well as key features of the chosen virus isolates, NS1 and NS2 protein sequences analyses (Figure S1 to Figure S4), identification of additional host factors 72 h post infection (Figure S5), molecular characterization of ADAR1 interaction with influenza NS1 protein and with dengue virus NS3 protein (Figure S6 to Figure S9), additional functional data in Huh-7 cells (Figure S10 and Figure S11). (DOC) [file ppat.1003440.s005.doc]

**Supporting Text 1**

**Cloning of influenza NS1 and NS2 cDNAs**

Open reading frames (ORFs) of NS1 and NS2 proteins from A/Puerto Rico/8/34 (H1N1), A/WSN/1933 TS61 (H1N1), A/Chicken/Scotland/1959 (H5N1), A/Chicken/Kurgan/3/2005 (H5N1), A/Vietnam/1194/2004 (H5N1), A/Chicken/Belgium/03 (H7N7), A/Equine/Prague/1/1956 (H7N7), A/Chicken/Guangdong/6/97 (H9N2), A/Duck/Australia/348/83 (H15N8) were amplified from plasmids coding for the corresponding cDNA genomic segments or from viral RNAs, using Gateway primers (containing 5’attB1.1 and 3’attB2.1 without ATG and stop codons). ORFs were cloned by *in vitro* recombination into donor vectors pDONR207, sequenced and stored into our viral ORFeotheque (all sequences are accessible through the viralORFeome database, [http://www.viralorfeome.com](http://www.viralorfeome.com/)).

**Key features of the chosen virus isolates**

*A/chicken/Kurgan/3/2005 (H5N1)*

Highly pathogenic strain in Molecular Genetics, Microbiology and Virology, 2011, Volume 26, Number 3, Pages 132-139 (no PMID available)

*A/Vietnam/1194/2004 (H5N1)*

Highly pathogenic avian influenza. Responsible for several deaths in East Asia in 2004 [1]

*A/chicken/Scotland/1959 (H5N1)*

Highly pathogenic avian influenza [2]

*A/chicken/Belgium/03 (H7N7)*

February 2003 outbreak in layer farms in the Netherlands. The outbreak ultimately spread to Belgium. The H7N7 2003 outbreak resulted in the destruction of 2.7 million birds in Belgium. An unexpectedly high number of transmissions of H7N7 virus to people directly involved in handling infected poultry as well as human-to-human transmission was observed during the outbreak [2]. 85 human cases were recorded, 1 of which was fatal.

*A/equine/Prague/1/1956 (H7N7)*

The equine-1 (H7N7) influenza virus is lethal in chickens and mice but not in horses. Induces an intense cytokine response in infected Balb/c mice but not in horses. The pathogenicity of H7N7 is believed to be dependent on the presence of multiple basic amino acids at the connecting peptide between HA1 and HA2 subunits.

*A/WSN/1933 TS61 (H1N1) and A/Puerto Rico/8/34 (H1N1)*

WSN strains were adapted from A/WS/33 virus, the first human influenza A isolate for neurovirulence in mice [3].

PR8 is a laboratory strain of influenza, known to be neurovirulent and lethal in mice. It is commonly used as a background vaccine for poultry and humans [4].

Laboratory mice are highly susceptible to infection with WSN and PR8 and severe disease or death is observed following administration of relatively low doses. In contrast, wild mice are resistant to even high doses of the same viral strains [5].

In 1946, Saslaw et al. intranasally inoculated rhesus macaques with the PR8 virus. No clinical signs of infection, including fever, anorexia, debility, or respiratory distress were observed; however, infected monkeys demonstrated leukopenia, primarily manifesting as a decrease in neutrophils, and developed neutralizing antibodies to the inoculating strain between 8 and 10 days post-infection. By contrast, in two monkeys inoculated by instillation of virus via syringe directly into the trachea, signs and symptoms consistent with influenza were observed, including listlessness and lethargy, facial flushing, and conjunctival injection. Symptoms persisted for two days, after which the animals returned to baseline. In these animals, neutropenia with reciprocal lymphocytosis was also observed [5].

*A/chicken/Guangdong/6/97 (H9N2)*

Low pathogenic avian influenza, not lethal in chicken [2]

*A/duck/Australia/348/83 (H15N8)*

No specific information available

**Phylogenetic analysis of selected NS1 and NS2 sequences**

To show that NS1 and NS2 proteins selected in this study are representative of the diversity in nature, we present their distribution along the phylogenetic trees constructed with all complete NS1 and NS2 protein sequences publicly available.

5,375 NS1 protein sequences and 2,722 NS2 protein sequences were retrieved from the Influenza Research Database [6]. We selected complete sequences independently of host specificity of the virus strains, excluding records with high similarity to 2009 pH1N1 sequences and duplicate sequences. The evolutionary history was inferred using the Minimum Evolution method (ME). The bootstrap consensus tree inferred from 100 replicates is taken to represent the evolutionary history of the taxa analyzed. Branches corresponding to partitions reproduced in less than 50% bootstrap replicates are collapsed. The tree is drawn to scale, with branch lengths in the same units as those of the evolutionary distances used to infer the phylogenetic tree. The evolutionary distances were computed using the Poisson correction method and are in the units of the number of amino acid substitutions per site. The ME tree was searched using the Close-Neighbor-Interchange algorithm at a search level of 0. The Neighbor-joining algorithm was used to generate the initial tree. All positions containing gaps and missing data were eliminated. Evolutionary analyses were conducted in MEGA5 [7].

Trees are drawn using a circle layout and replacing FASTA sequence headers by a number, except for the 9 NS1 and the 9 NS2 sequences selected for this study. In Figures S1 and S2, are represented the distributions of these proteins on their respective phylogenetic trees.


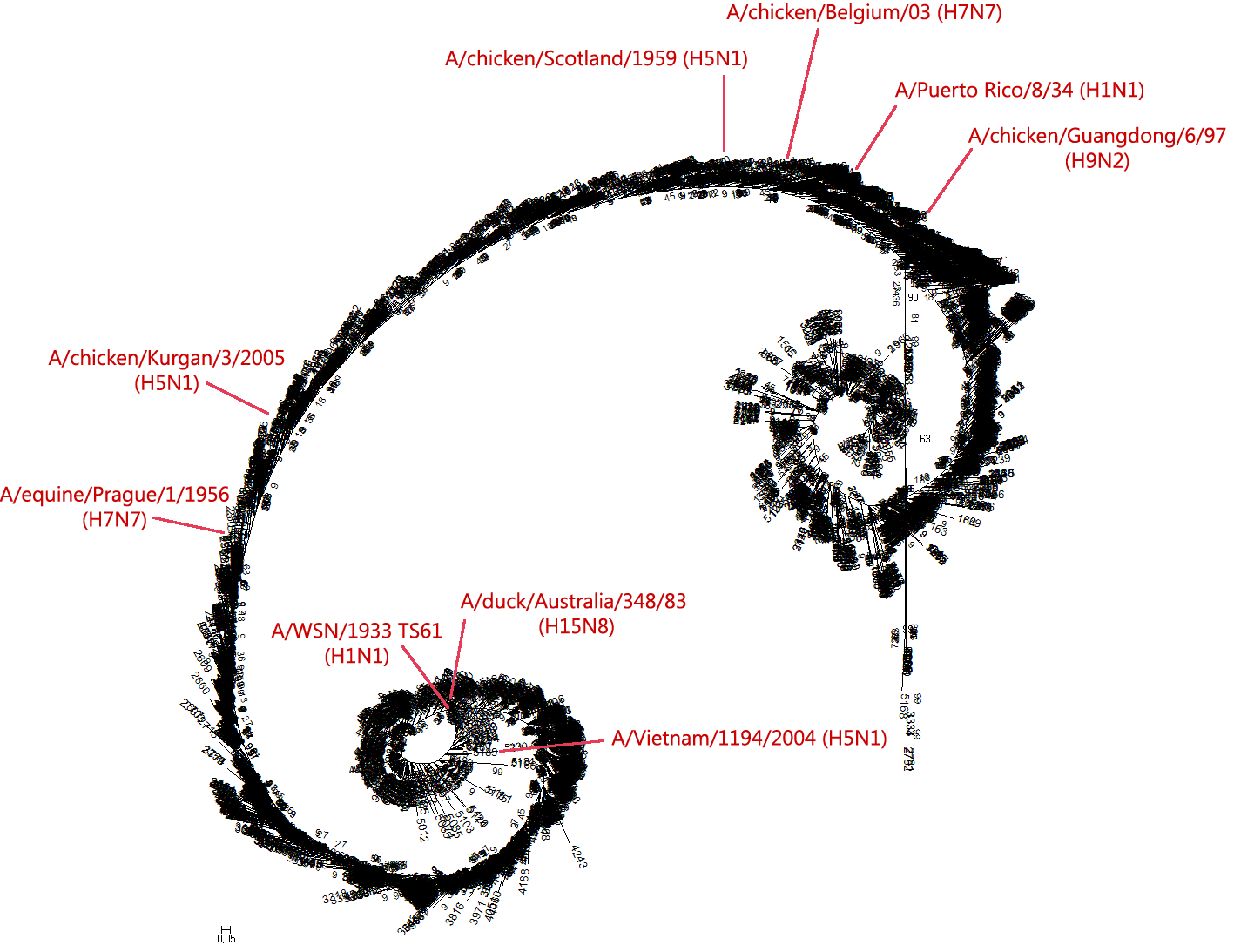


**Figure S1: Phylogenic tree of the influenza virus strains based on all the NS1 protein sequences publicly available.** Only the 9 NS1 proteins used in this study are clearly labeled.

**
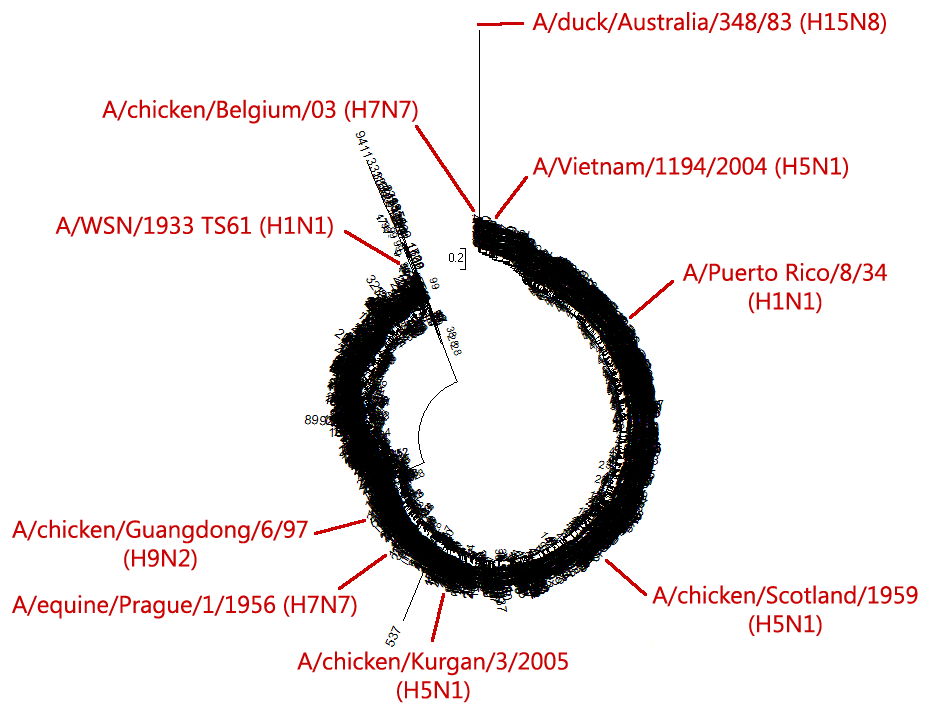
**

**Figure S2: Phylogenic tree of the influenza virus strains based on all the NS2 protein sequences publicly available.** Only the 9 NS2 proteins used in this study are clearly labeled.

Studied NS1 and NS2 proteins are regularly distributed on their phylogenetic trees from end to end.

**Alignments of NS1 and NS2 sequences**


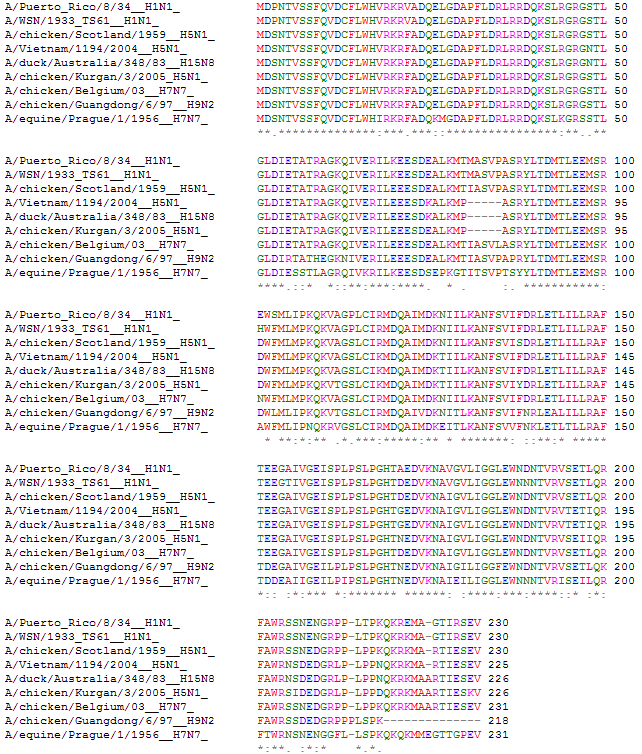


**Figure S3:** NS1 sequences alignement using ClustalW at the Pôle Bioinformatique Lyonnais.


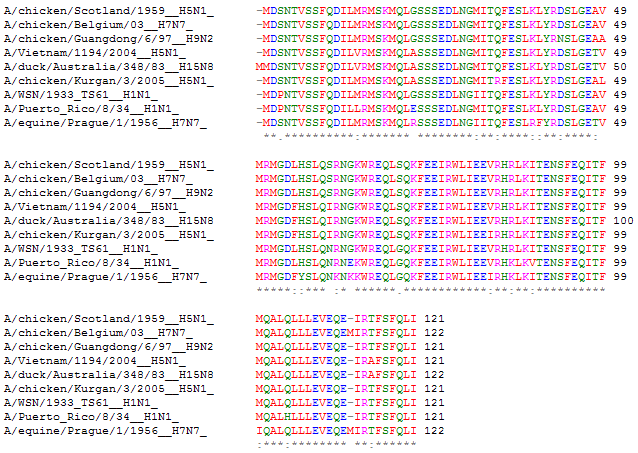


**Figure S4:** NS2 sequences alignement using ClustalW at the Pôle Bioinformatique Lyonnais.

**Human interactome dataset**

Since no database contains the complete human interactome, the dataset used in this study has been reconstructed from several public databases (VirHostNet database [8]). We have selected sources providing physical interactions, thus avoiding predicted or genetic interactions. The interactome dataset is currently composed of 10,707 proteins and 55,681 interactions. It has been studied in details in a complete interactomic study recently published by our team [9]. It has a network resilience typical of scale-free architecture, is small-world and diassortative. Its network properties fit with those of other eukaryotic interactomes described in the literature.

**Network metrics**

Mean degree of NS1 and NS2 interactors is 24.60 vs. 10.43 for the human interactome (calculated from the VirHostNet human protein-protein interaction network). Degree distribution comparison (NS1 and NS2 interactors versus all human proteins): NS1 and NS2 interactors greater than all human proteins (Wilcoxon/U-test p-value: 1.443 10^-15^).

NS1 and NS2 interactors adjusted mean betweenness is 0.0004286151 vs. 0.0001300057 for the human interactome (computed from the VirHostNet human protein-protein interaction network). Betweenness distribution comparison (NS1 and NS2 interactors versus all human proteins): NS1 and NS2 interactors greater than all human proteins (Wilcoxon/U-test p-value: 5.995 10^-15^).

**Cell viability assay**

Alamar Blue Cell viability assay was used following manufacturer’s instructions (resazurin assay, Lifetechnologies).

**Quantification of gene silencing by qRT-PCR**

Total RNA was extracted by using NucleoSpin RNA II (Macherey-Nagel, Germany) according to the manufacturer’s instructions. mRNA levels were determined by using the 2x green DYE Master Mix (P.J.K, Germany). GAPDH mRNA was used for normalization of input RNA. RT-PCR data were analyzed by using the ΔCT method described previously (Livak and Schmittgen, 2001). Reactions were performed on an ABI PRISM 7000 sequence detection system (Applied Biosystems, Germany) using the following program: 95°C for 10 min and 40 cycles as follows: 95°C for 30 s, 55°C for 1 min, and 72°C for 1 min. Primer sequences are given in Table S4.

**Analysis of enrichment for relevant host factors**

Seven relevant host factors among 79 proteins have been identified. We have statistically compared this ratio (7/79) to the ratios obtained in other genetic screens for influenza virus replication [10]. The p-value was assessed using an exact Fisher test.

| **Screen** | **Nb genes tested** | **Nb relevant host factors identified** | **%** | **P-value**  **(is there a significant enrichment in our screen?)** |
| --- | --- | --- | --- | --- |
| Our screen | 79 | 7 | 8.86% |  |
|  |  |  |  |  |
| Brass [11] | 17877 | 133 | 0.75% | 2.73 10^-6^ |
| König [12] | 19628 | 295 | 1.5% | 2.1 10^-4^ |
| Shapira [13] | 1745 | 242 | 13.9% | 0.9315 |
| Hao [14] | 10278 | 78 | 0.76% | 3.51 10^-6^ |
| Ward [15] | 21125 | 235 | 1.1% | 3.33 10^-5^ |

Our screen shows a significant enrichment in host factors compared to the other screens (p-value <= 2.1 10^-4^) expect Shapira’s. In their screen, Shapira and colleagues used interactomic, transcriptomic and functional filters to select a subset of 1745 genes that were further submitted to genetic screens (siRNAs). We obtained a ratio similar to that of Shapira, highlighting the relevance of such filters.

**Pro-viral host factors identified 72h post infection**

A549 cells were transfected with the indicated siRNAs, infected with influenza A H1N1 and the neuraminidase activity (NA) was measured in the supernatant 72h post infection. Values represent the mean +/- standard deviation (triplicates). ATP6V1G1 is a control pro-viral host factor. GMEB1 was finally excluded because of poor silencing efficiency induced by both siRNAs.


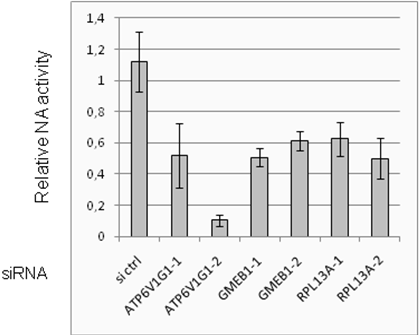


**Figure S5**

**Plaque assay**

Serial dilutions of supernatants were prepared in DMEM containing 1 µg/ml TPCK-trypsin then added to MDCK cells in 6 well plates. Plates were incubated 1h30 at 37 °C, washed with serum-free medium and final overlay was performed with low gelling temperature agarose (SeaKem ME agarose, Lonza) in MEM (Life Technologies) containing 1 µg/ml TPCK-trypsin. Cells were cultured at 37 °C for 2-3 days, fixed in 10 % formaldehyde (Sigma) then stained with 0.3 % crystal violet (Sigma) and plaques were counted.

**IFN-β secretion assay**

Supernatants of siRNA-transfected A549 cells were collected after 48h infection (MOI 0.5), conserved at -80 °C and assayed for IFN-β secretion using VeriKine Human IFN-β ELISA kit (PBL biomedical laboratories).

**Mapping of a conserved 47 amino acid residues region in ADAR1**

DRBDs from ADAR1, SON, ILF3, STRBP, STAU1 were aligned using ClustalW at the Pôle Bioinformatique Lyonnais.


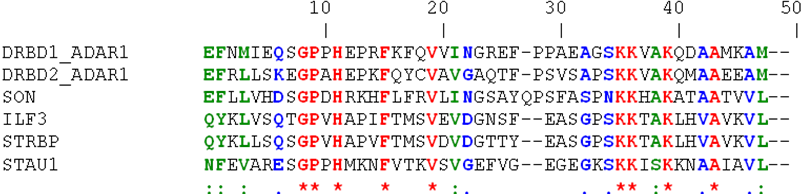


**Figure S6**

**ADAR1 constructs and siRNAs**

ADAR1 full-length was purchased from Life Technologies. ADAR1 truncated constructs were amplified by PCR (KOD polymerase, Novagen) and cloned into pDONR207 (Life Technologies) using a recombinational cloning system (Gateway, Life Technologies). 5′ ends of forward primers were fused to Gateway cloning sequence 5′-GGGGACAACTTTGTACAAAAAAGTTGGCATG-3′ and reverse primers were fused to 5′-GGGGACAACTTTGTACAAGAAAGTTGGCTA-3′. ΔDRBD1, ΔDRBD2, ΔDRBD3 forward primer sequences were respectively 5′-GTCACCACACTGCTTGAG-3′, 5′-CCTGTGGGTGGCCTTTTG-3′ and 5′-ATTGGGGAGAACGAGAAG-3′, while reverse primer was 5′-TACTGGGCAGAGATA-3′. Amplified constructs correspond to AA 615-1226 (ΔDRBD1), 726-1226 (ΔDRBD2) and 792-1226 (ΔDRBD3). DRBD1 peptide coding sequence was amplified using 5′-GAGTTCAACATGATAGAGCAGA-3′ and 5′-CATGGCTTTCATAGCTGCAT-3′ forward and reverse primers respectively, flanked in 5′ with the Gateway cloning sequence.

Stealth siRNAs were purchased from Life Technologies and transfected according to manufacturer instructions. si1 ADAR: ADARHSS100168; si2 ADAR: ADARHSS100169; Control siRNA : med GC: 12935300. An additional anti-ADAR1 siRNA with the following sequence 5´CGCAGAGUUCCUCACCUGUA is a custom sequence from MWG.

**Editing reporter plasmid** **catalytically inactive mutant ADAR1**

For RNA editing assay, we first generated an editing reporter plasmid from a plasmid encoding the Renilla luciferase (RLUC) upstream the Firefly luciferase (FLUC) under the control of the human CMV promoter. The two sequences are separated by a short spacer allowing translation of the Firefly luciferase by ribosomal reinitiation. The reporter was constructed by replacement of this reinitiation region by the Hepatitis Delta virus (HDV) minimal sequence edited by ADAR1 [16]. Briefly, RLUC was amplified by using primers 1 and 2 and FLUC by using primers 3 and 4 (KOD polymerase, Novagen). Overlapping fragments 1-2 and 3-4 were mixed and used as templates for a new PCR round using primers 1 and 4. This amplified RLUC-FLUC fusion fragment separated by the editing sequence was cloned between *Xho1* and *Sph1* sites to generate the final construct.

Primer 1: 5'-GAATTC*CTCGAG*GAACTGG-3' (forward)

Primer 2: 5'-atgggatgcgtatatcctatggTTGTTCATTTTTGAGAACTCGCTC-3' (reverse).

Primer 3: 5'-ggatatacgcatcccatgggtcGACGCCAAAAACATAAAGAAAGGCCC-3' (forward)

Primer 4: 5'- ATCTCTG*GCATGC*GAGAATCTGACGC-3' (reverse)

The catalytically inactive mutant ADAR1 has been described elsewhere [17]. ADAR1 E912A mutation was performed using the Gene Art site-directed mutagenesis system on pCIneo3xFlag ADAR1 plasmid with primers E912A (+) and E912A (-):

E912A(+) : 5’- AATGACTGCCATGCAGCAATAATCTCCCGGAGA -3’

E912A(-) : 5’-TCTCCGGGAGATTATTGCTGCATGGCAGTCATT-3’

**Contribution of RNA to the interaction between ADAR1 and viral proteins**

Both ADAR1-NS1 and ADAR1-NS3 interactions are mediated by the RNA-binding domain of the two proteins. The involvement of RNA in these interactions was addressed in GST pull-down experiments. Following GST pull-down, beads were treated with RNAse A in 100 mM NaCl (allowing cleavage of single-stranded and double-stranded RNA). The amount of ADAR1 bound to beads and released in the supernatant was then detected. For both interactions, a detectable amount of ADAR1 was released in the supernatant when beads were treated with RNAse A, indicating that RNA can at least be partially involved in ADAR1-NS1 and ADAR1-NS3 interactions. Contribution of RNA to the interaction between ADAR1 and NS1 was also addressed by GST pull-down using a mutated NS1 (R38A K41) that lacks double-stranded RNA-binding activity. This mutant still interacts with ADAR1 albeit with less intensity, indicating that RNA is not strictly requested for NS1 interaction with ADAR1.


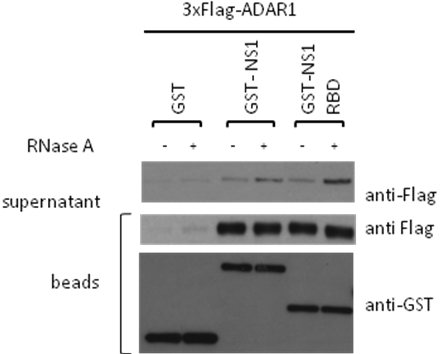


**Figure S7**

Marginal involvement of RNA in influenza virus NS1-ADAR1 and NS1 RBD-ADAR1 interaction. NS1-ADAR1 and NS1 RBD-ADAR1 complexes were treated with 0 (-) or 2 µg of RNase A (+). Glutathione bound proteins and supernatants were analyzed by immunoblotting using antibodies against GST or Flag.


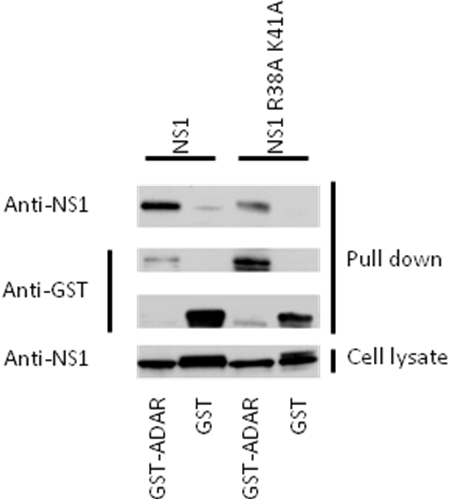


**Figure S8**

RNA is not strictly required for NS1 interaction with ADAR1.


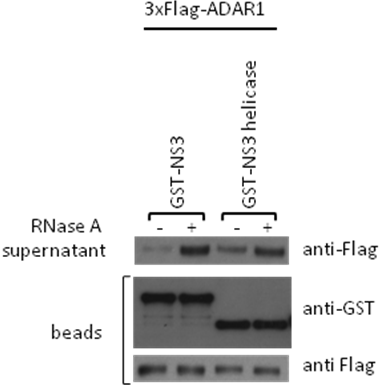


**Figure S9**

Marginal involvement of RNA in dengue NS3-ADAR1 and dengue NS3 helicase-ADAR1 interaction. NS3-ADAR1 and NS3 helicase-ADAR1 complexes were treated with 0 (-) or 2 µg of RNase A (+). Glutathione bound proteins and supernatants were analyzed by immunoblotting using antibodies against GST or Flag.

**ADAR1 silencing in Huh-7 cells**


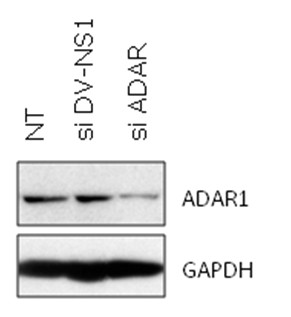


**Figure S10**

ADAR1 expression in Huh-7 following transfection of ADAR1-specific siRNAs (control dengue virus NS1 specific-siRNA, DV-NS1). 72h post-transfection, cells were infected with a DV Renilla reporter at an MOI of 0.5 and ADAR1 was detected by immunoblotting 48h later.

**Impact of ADAR1 silencing on Huh-7 cells stably expressing the dengue virus replicon**

10^6^ Huh-7 cells stably expressing the subgenomic replicon dengue virus NS1-NS5 with Renilla reporter were electroporated with 0.25 nmol siRNAs. 72h post-electroporation, cells were lyzed, Renilla RLU was measured and values normalized to the non-targeting control (siRNA directed against GFP).


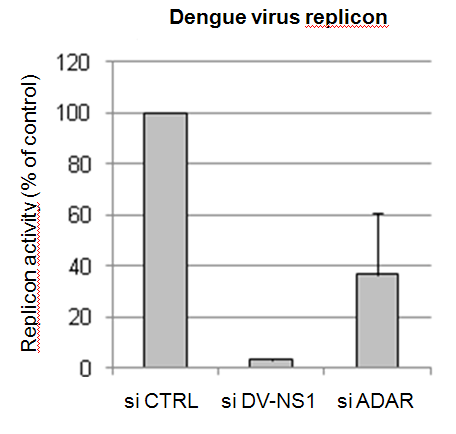


**Figure S11**

A strong decrease of replicon activity was observed in Huh-7 after silencing of ADAR1 expression by RNA interference.

**Table S1. Origin of NS1 and NS2 proteins used in this study. Sequences are available through ViralORFeome database, using indicated clone identification number.**

**Table S2. NS1 and NS2 interactors expressed in lung and trachea.**

**Table S3. Complete list of NS1 and NS2 interactors.**

Y2H: interactors identified in this study by yeast two-hybrid screening and arrays. Literature: interactors mined by text mining.

**Table S4. List of host proteins identified in Y2H screens and their impact on virus replication and type I interferon β production.** Data are normalized to controls. NT: not tested. ND: not detectable. The control value in this experiment is 2 pg/ml. The detection limit corresponds to 1.2 pg/ml. Therefore we cannot conclude that a gene is a positive regulator when its silencing leads to a not-detectable level of IFN.

**References**

1. Nicolson C, Major D, Wood JM, Robertson JS (2005) Generation of influenza vaccine viruses on Vero cells by reverse genetics: an H5N1 candidate vaccine strain produced under a quality system. Vaccine 23: 2943-2952.

2. Lupiani B, Reddy SM (2009) The history of avian influenza. Comp Immunol Microbiol Infect Dis 32: 311-323.

3. Steinhauer DA (1999) Role of hemagglutinin cleavage for the pathogenicity of influenza virus. Virology 258: 1-20.

4. Zhang W, Xue T, Wu X, Zhang P, Zhao G, et al. (2011) Increase in viral yield in eggs and MDCK cells of reassortant H5N1 vaccine candidate viruses caused by insertion of 38 amino acids into the NA stalk. Vaccine 29: 8032-8041.

5. Bouvier NM, Lowen AC (2010) Animal models for Influenza virus pathogenesis and transmission. Viruses 2: 1530-1563.

6. Squires RB, Noronha J, Hunt V, Garcia-Sastre A, Macken C, et al. (2012) Influenza research database: an integrated bioinformatics resource for influenza research and surveillance. Influenza Other Respi Viruses 6: 404-416.

7. Tamura K, Peterson D, Peterson N, Stecher G, Nei M, et al. (2011) MEGA5: molecular evolutionary genetics analysis using maximum likelihood, evolutionary distance, and maximum parsimony methods. Mol Biol Evol 28: 2731-2739.

8. Navratil V, de Chassey B, Meyniel L, Delmotte S, Gautier C, et al. (2009) VirHostNet: a knowledge base for the management and the analysis of proteome-wide virus-host interaction networks. Nucleic Acids Res 37: D661-668.

9. Meyniel-Schicklin L, de Chassey B, Andre P, Lotteau V (2012) Viruses and interactomes in translation. Mol Cell Proteomics 11: M111 014738.

10. de Chassey B, Meyniel-Schicklin L, Aublin-Gex A, Andre P, Lotteau V (2012) Genetic screens for the control of influenza virus replication: from meta-analysis to drug discovery. Mol Biosyst 8: 1297-1303.

11. Brass AL, Huang IC, Benita Y, John SP, Krishnan MN, et al. (2009) The IFITM proteins mediate cellular resistance to influenza A H1N1 virus, West Nile virus, and dengue virus. Cell 139: 1243-1254.

12. Konig R, Stertz S, Zhou Y, Inoue A, Hoffmann HH, et al. (2010) Human host factors required for influenza virus replication. Nature 463: 813-817.

13. Shapira SD, Gat-Viks I, Shum BO, Dricot A, de Grace MM, et al. (2009) A physical and regulatory map of host-influenza interactions reveals pathways in H1N1 infection. Cell 139: 1255-1267.

14. Hao L, Sakurai A, Watanabe T, Sorensen E, Nidom CA, et al. (2008) Drosophila RNAi screen identifies host genes important for influenza virus replication. Nature 454: 890-893.

15. Ward SE, Kim HS, Komurov K, Mendiratta S, Tsai PL, et al. (2012) Host modulators of H1N1 cytopathogenicity. PLoS One 7: e39284.

16. Sato S, Wong SK, Lazinski DW (2001) Hepatitis delta virus minimal substrates competent for editing by ADAR1 and ADAR2. J Virol 75: 8547-8555.

17. Heale BS, Keegan LP, McGurk L, Michlewski G, Brindle J, et al. (2009) Editing independent effects of ADARs on the miRNA/siRNA pathways. EMBO J 28: 3145-3156.
